# Long noncoding RNAs as novel predictors of survival in human cancer: a systematic review and meta-analysis

**DOI:** 10.1186/s12943-016-0535-1

**Published:** 2016-06-28

**Authors:** Stylianos Serghiou, Aikaterini Kyriakopoulou, John P. A. Ioannidis

**Affiliations:** St. John’s Hospital, Livingston, EH54 6PP UK; College of Medicine and Veterinary Medicine, University of Edinburgh, Edinburgh, UK; University Hospital of North Durham, North Rd, Durham, DH1 5TW UK; Stanford Prevention Research Center, Department of Medicine, Stanford University School of Medicine Stanford, Stanford, CA 94305 USA; Department of Health Research and Policy, Stanford University School of Medicine, Stanford, CA 94305 USA; Department of Statistics, Stanford University School of Humanities and Sciences, Stanford, CA 94305 USA; Meta-Research Innovation Center at Stanford (METRICS), Stanford University, 1265 Welch Rd, MSOB X306, Stanford, CA 94305 USA

**Keywords:** LncRNA, Cancer, Cancer biomarkers, Prognosis, Survival analysis, Excess significance, Small-study effects, Selective reporting biases

## Abstract

**Background:**

Expression of various long noncoding RNAs (lncRNAs) may affect cancer prognosis. Here, we aim to gather and examine all evidence on the potential role of lncRNAs as novel predictors of survival in human cancer.

**Methods:**

We systematically searched through PubMed, to identify all published studies reporting on the association between any individual lncRNA or group of lncRNAs with prognosis in human cancer (death or other clinical outcomes). Where appropriate, we then performed quantitative synthesis of those results using meta-analytic methods to identify the true effect size of lncRNAs on cancer prognosis. The reliability of those results was then examined using measures of heterogeneity and testing for selective reporting biases.

**Results:**

Three hundred ninety-two studies were screened to eventually identify 111 eligible studies on 127 datasets. In total, these represented 16,754 independent participants pertaining to 53 individual and 6 grouped lncRNAs within a total of 19 cancer sites. Overall, 83 % of the studies we identified addressed overall survival and 32 % of the studies addressed recurrence-free survival. For overall survival, 96 % (88/92) of studies identified a statistically significant association of lncRNA expression to prognosis. Meta-analysis of 6 out of 7 lncRNAs for which three or more studies were available, identified statistically significant associations with overall survival. The lncRNA HOTAIR was by far the most broadly studied lncRNA (*n* = 29; of 111 studies) and featured a summary hazard ratio (HR) of 2.22 (95 % confidence interval (CI), 1.86–2.65) with modest heterogeneity (I^2^ = 49 %; 95 % CI, 14–79 %). Prominent excess significance was demonstrated across all meta-analyses (*p*-value = 0.0003), raising the possibility of substantial selective reporting biases.

**Conclusions:**

Multiple lncRNAs have been shown to be strongly associated with prognosis in diverse cancers, but substantial bias cannot be excluded in this field and larger studies are needed to understand whether these prognostic information may eventually be useful.

**Electronic supplementary material:**

The online version of this article (doi:10.1186/s12943-016-0535-1) contains supplementary material, which is available to authorized users.

## Background

Non-coding RNAs (ncRNAs) have been proposed in the last decade as regulators of cancer pathways and biomarkers of cancer outcomes [1–4]. Potentially informative biomarkers based on ncRNAs include microRNAs (miRs) [5] and the larger long non-coding RNAs (lncRNAs). NcRNAs were up to recently disregarded as ‘junk’ and despite constituting the large majority of RNAs being transcribed, their role in normal development and cellular physiology in health and disease is only recently becoming apparent [2, 6, 7].

LncRNAs refer to any ncRNA consisting of more than 200 nucleotides. They are functionally heterogeneous molecules [6, 8], themselves sub-classified into large intergenic non-coding RNAs (lincRNA), transcribed ultraconserved regions (T-UCRs) and many others [2]. Of an estimated putative 140,000 different ncRNAs in total [9], lncRNAs are estimated to constitute proportionally the largest class, with the most comprehensive approach to date confirming 58,648 expressed lncRNAs [10]. Even though the function of lncRNAs is still being debated [11], certain lncRNAs have been implicated in functions related to regulation of gene expression in health and disease [2, 6–8, 12–15]. Well-studied examples include the lncRNA *Xist*, which initiates X-chromosome inactivation in female cells by recruiting repressive complexes to the X-chromosome under inactivation [16–18] and *H19*, which has been shown to play a significant role in genomic imprinting [19, 20].

Of particular interest however is, that it is now clear that lncRNAs are major players in tumorigenesis [7–9, 21–23]. In this context, the most well studied lncRNA is HOTAIR (HOmeobox (HOX) Transcript AntIsense RNA), which has been shown to recruit the PRC2 (Polycomb Repressive Complex 2) complex and eventually lead to epigenetic silencing of metastasis suppressor genes [2, 24].

More than 20 meta-analyses studying the role of lncRNAs in cancer prognosis have been published so far, all within the past 2 years. All of these studied a single lncRNA, either in relation to a specific cancer or to any cancer. The two most studied lncRNAs are MALAT1 and HOTAIR, which have been the subject of 10 and 7 meta-analyses respectively. The latest meta-analysis on MALAT1 for all cancer types showed that its upregulation is statistically significantly associated with poor overall survival (pooled hazard ratio [HR], 2.14; 95 % CI, 1.74–2.64) with low between-study heterogeneity (I^2^,  4.3 %; *p*-value  = 0.399), on the basis of 9 studies [25]. The results were similar to the latest meta-analysis of HOTAIR (HR, 2.33; 95 % CI, 1.77–3.09), but with significant between-study heterogeneity (Cochran’s Q-test *p*-value = 0.016), on the basis of 16 studies [26]. Interestingly, all meta-analyses published so far have been produced by Chinese groups and all identified a statistically significant association of all lncRNAs studied to prognosis in cancer. However, no systematic review and meta-analysis to-date has identified all lncRNAs studied in the context of cancer and to what extent these might be of prognostic significance.

In this paper, we aimed to examine the potential role of all lncRNAs ever investigated in the context of cancer survival prediction, as novel predictors of survival in human cancer. We utilized a field-wide meta-analysis approach [27] to systematically identify and examine all published papers trying to associate lncRNAs to prognosis in human cancer, and to quantitatively synthesize data directly related to prognosis wherever three or more studies on an lncRNA had been done. 

## Methods

### Systematic review

This report has been structured on the basis of PRISMA [28]. 

#### Eligibility criteria

We considered published reports of a prospective or retrospective study design that had explored the association of any single or combination of stated lncRNAs to any of the following types of survival analysis: disease-specific survival (DSS, duration of time from the day of diagnosis to the day of death due to cancer); metastasis-free survival (MFS, duration of time from day of diagnosis to the day of diagnosing a metastatic event); overall/cumulative survival (OS, duration of time from day of diagnosis to the day of death due to any cause); progression/event/disease-free survival (PFS, duration of time from day of first treatment to the day evidence of cancer progression are identified or the patient dies of any cause); and recurrence-free survival (RFS, duration of time from day of cure from cancer to the day evidence of cancer progression/recurrence is identified). Survival analyses measuring different types of survival were treated separately at all times. Studies describing the association of individual or groups of lncRNAs with clinicopathologic variables (e.g. Stage, Grade, Distant metastasis, etc.), without specifically examining associations to any of the aforementioned survival analyses, were excluded. We likewise excluded cross-sectional studies and studies concerning genetic alterations of an lncRNA (e.g. polymorphisms or methylation patterns). Any kind of quantitative lncRNA analysis (quantitative real time–PCR, *in situ* hybridization) was eligible.

For meta-analysis eligibility, a study had to also provide the effect size and confidence interval for the association of an individual or group of lncRNAs with any of the above survival outcomes, or report information through which this effect size and confidence interval could be calculated [29, 30]. Wherever the same cohort had published more than one overlapping analysis, we only used the most encompassing data (for example, the classification of glioma would be preferred over glioblastoma multiforme). Two reviewers (S. Serghiou and A. Kyriakopoulou) identified eligible studies, and any contested articles were adjudicated by a third reviewer (J. P. A. Ioannidis).

#### Information sources

We systematically searched PubMed (1950 to September, 2015) for studies of any language that analyzed associations between lncRNAs and prognosis in human cancer. Our search strategy was developed in consideration of previous recommendations [30] and used the clinical queries prognosis filter, which has been reported to have an average estimated sensitivity of 92 % for detecting articles related to prognosis [5, 31]. Our search term was: (Prognosis/Broad [filter]) AND ((lncRNA OR “lnc RNA” OR “long noncoding ribonucleic acid” OR “long noncoding RNA” OR “long non-coding ribonucleic acid” OR “long intergenic noncoding RNA” OR “long intergenic non-coding RNA” OR “long non-coding RNA” OR “long ncRNA” OR “lincRNA” OR “linc RNA”) AND (cancer OR carcinoma OR tumor OR neoplas* OR tumour OR malignan* OR metastat* OR metastas* OR leukemia OR leukaemia OR lymphoma OR recurren* OR “lymph node” OR response) AND (Humans[Mesh] AND English[lang])). The search was last updated to include articles published through September 26, 2015.

#### Study selection

We used the programming language R [32] to remove duplicate records. Title and abstract were screened to identify relevant articles. The full manuscript of the relevant articles was screened against our eligibility criteria. Any uncertainties were resolved by consensus with JPA. Data were collected by two reviewers (SS, AK) and saved in a pre-designed extraction form on Google Sheets. Where information was ambiguous (such as, for example, mentioning multiple types of lncRNA quantification methods but not clarifying which one of those was used to provide the quantities utilized in the survival analysis), this was labelled as ‘unclear’. An attempt was made to contact the authors when information was clearly logically inconsistent, as in for example quoting a hazard ratio (HR) outside the confidence interval (CI), but none replied. In one paper, the lncRNA expression level [33] was subdivided into low versus medium versus high; for this paper we only extracted the comparison between low versus high expression levels. The following data were extracted for all articles following the CHARMS checklist [34]: title; authors; year of publication; journal of publication; groupings (i.e. whether lncRNAs were studied one by one or in groups); what lncRNAs were studied; whether an agnostic approach to identifying the studied lncRNAs was used (where an agnostic approach would be one assuming no prior knowledge regarding the choice of lncRNA to be studied); cancer site (e.g. brain) and cancer subtype (e.g. glioblastoma multiforme); whether a paper reported clinicopathologic data of its sample and which ones; whether an attempt of associating those clinicopathologic data to lncRNAs was made and for which ones; whether an attempt of associating clinicopathologic data to prognosis was made and for which ones; whether an attempt was made to explain the clinical outcomes using non-clinical studies (*in vivo, in vitro*); the types of survival analyses used (as above); type of study design (prospective cohort, retrospective cohort, unreported); means of lncRNA quantitative analysis (qRT–PCR, qPCR, *in situ* hybridization (ISH), other); and whether the paper tried to make any non-clinical associations of the identified lncRNAs to cancer *in vitro*. For eligible articles we further extracted: country and city of origin of the study cohort, period of sample recruitment, range of sample ages, mean/median age with confidence interval, the population type (general population, non-general population (e.g. veterans), unreported), stage of cancer upon initial patient presentation, sample size, means of tissue preservation (frozen, paraffin-embedded, both, other), any and what preoperative treatment was given, the total number of lncRNAs studied, the type of metric the paper used to characterize their results (hazard ratio, relative risk, odds ratio, *p*-value), type of analysis (i.e. univariable or multivariable), lncRNA quantity cut-off and its unit (i.e. the threshold based on which lncRNA expression was deemed upregulated or downregulated by the study), the sample size of each comparison group, the minimum and maximum participant follow-up time, the number of censored participants throughout follow-up and whether this was explicitly stated or read off the Kaplan-Meier curves, the HR and its CI (provided or inferred, e.g. from *p*-values and HR point estimates), the *p*-value and whether this was statistically significant at *p* < 0.05 and whether an attempt to validate the reported results was made, and if so, what type of validation method was used (internal or external). For eligibility for meta-analysis, enough information to extract or calculate the natural logarithm of the hazard ratio and its variance must have been provided.

Whenever multiple datasets were combined into a single dataset to study a specific lncRNA, we only extracted the *summary* HR, rather than extracting the HR respective to each constitutive dataset. If multiple datasets were assessed within the same study without being combined into a single dataset, we extracted the HR respective to each dataset, as they represent separate estimates. Where both the log-rank and Breslow tests were reported, only the log-rank was extracted. No cohort was used more than once and effect sizes describing a broader class of cancer (e.g. glioma) were preferred over subclassifications of that (e.g. glioblastoma multiforme). Three studies reported effect sizes that were excluded from further consideration because the quoted HRs contradicted the text [35] or they were either outside the CI or could not have possibly led to the quoted CI [36, 37]; this led to complete exclusion of two out of these three studies [35, 37]. Our database can be freely accessed here: https://goo.gl/EjCDAp.

#### Risk of bias in individual studies

Risk of bias in individual studies was assessed on the basis of the framework of assessing internal validity of articles dealing with prognosis [30, 38] and recommendations regarding reporting of biomarker studies [39, 40].

### Meta-analysis

#### Summary measures and synthesis of results

We meta-analyzed data on lncRNAs for which three or more estimates of their effect on a specific survival outcome were available. Therefore, meta-analyses were only done for OS and RFS. Effect sizes for OS and RFS were meta-analyzed separately. Our principal summary measure was the summary HR. Standard errors were calculated using: ln (upper limit of CI/lower limit of CI)/(2 × 1.96). Estimates were synthesized using a random-effects model and estimated using the restricted maximum-likelihood ratio method. As previously described [27], four meta-analyses were done for each of: (1) multivariable data, (2) univariable data, (3) multivariable data combined with univariable data whenever multivariable data were unavailable (preferentially multivariable) and (4) univariable data combined with multivariable data whenever univariable data were unavailable (preferentially univariable). Given the similarity between the estimates of all four types of meta-analysis and the importance of multivariable modelling in prognostic studies, this report only quotes the estimates of the ‘preferentially multivariable’ meta-analysis; the rest can be found in Additional file 1: Table S2. For each estimate we provide the effect size and 95 % CI. Heterogeneity was analyzed using the Q and I^2^ statistics and the 95 % CI of I^2^ was also calculated [41, 42]. These analyses were done using R and the package metafor 1.9-8 [43]. Data were combined for each type of lncRNA regardless of cancer type. Wherever an lncRNA had been analyzed three or more times for one or more specific cancer type, a *post hoc* subgroup analysis per cancer type was done for that lncRNA.

#### Risk of bias across studies

Risk of publication bias is a significant concern in prognostic studies [30]. We explored excess significance for factors reported by at least 3 studies [44]. Briefly, for every meta-analyzed risk factor we compare the number of observed significant results (O) at α = 0.05, to the number of expected significant results (E), where E = sum of power of each study within a specific meta-analysis. Power was calculated taking as plausible effect for the risk factor the effect seen in the most precise study (lowest standard error). The difference between O and E was assessed using a two-tailed binomial test, with α = 0.1, as previously suggested [45]. O and E were also summed and compared across all meta-analyses.

## Results

### Literature search and description of studies

**Fig. 1:**
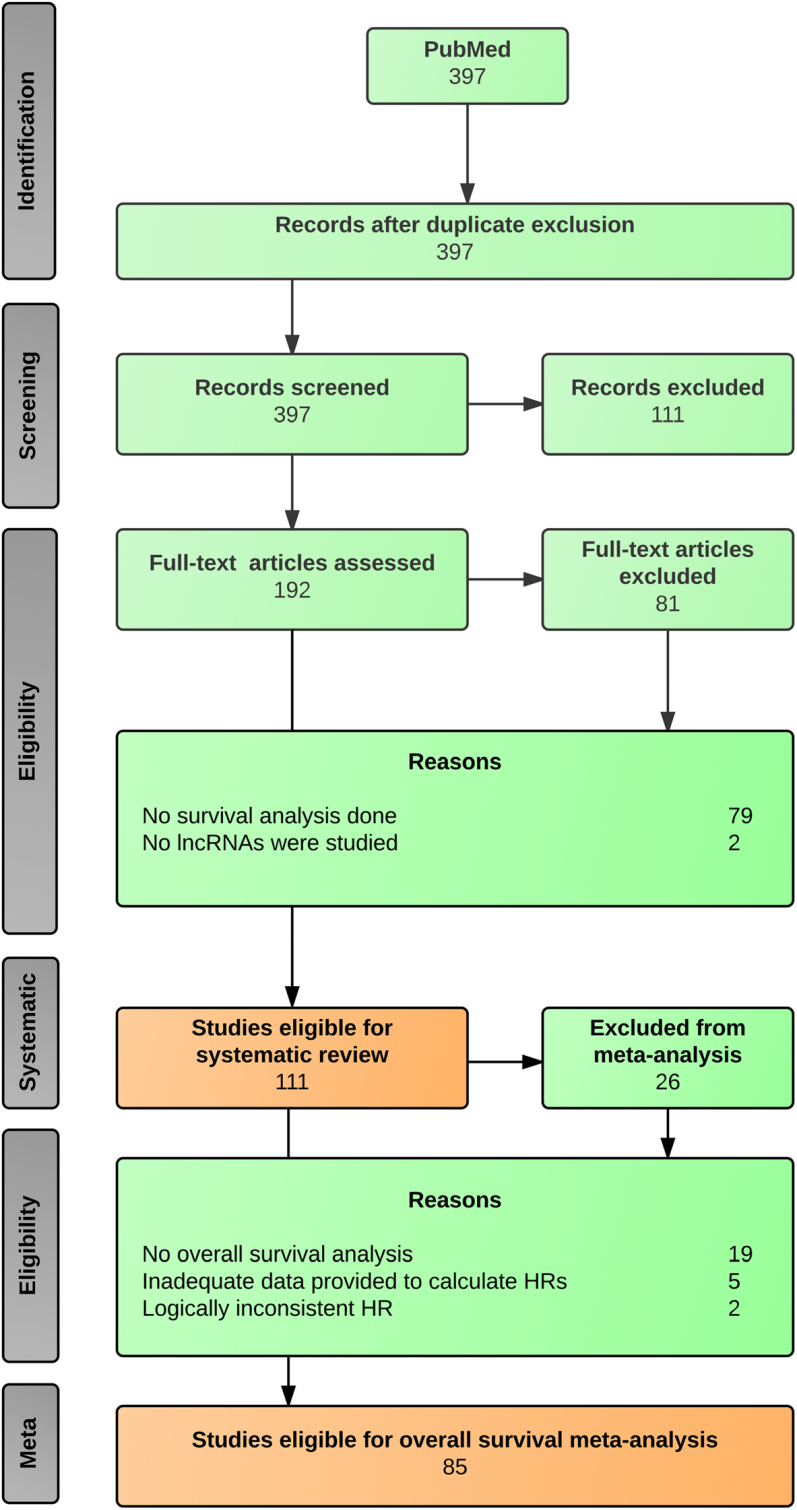
A flow diagram demonstrating the study selection process. Out of 397 identified records, 111 were chosen for systematic review and 85 for overall survival meta-analysis. Duplicate exclusion refers to the process of asserting that each paper is only represented once within our set of records. Initially, 111 records with titles seemingly irrelevant to the objectives of our study were excluded, following which another 81 records were excluded after reading through the remaining 192 papers, for the reasons identified within the diagram. This process led to the identification of 111 papers eligible for systematic review. We then applied our meta-analysis eligibility criteria to these papers, on the basis of which 26 were excluded, for the reasons identified in the diagram. This process led to the identification of 85 papers eligible for meta-analysis

We initially identified 397 records, from which 286 were excluded (Fig. 1), leaving us with 111 studies eligible for systematic review (Additional file 2), of which 85 were also eligible for meta-analysis. The 111 studies utilized 127 datasets to produce their analyses (four studies utilized two datasets, three studies utilized three datasets and two studies utilized four datasets). No new studies were imported through reference checking.

Of 127 identified datasets, only 2 were reported to represent a prospective cohort; of the rest, 19 were reported to represent a retrospective cohort and there were no relevant information for the remaining 106 datasets. No report specified what type of population their samples came from and for 113/127 datasets we have no information as to what sampling method was used to obtain the sample. For the remaining datasets, consecutive sampling was stated to have been used in 5 and random sampling in 4 datasets; 5 datasets were based on all patients ever seen by the clinic. Sampling method was disproportionately frequently reported for studies coming from the USA (4/9). A total of 94/127 datasets came from Asia (78 from China), followed by Europe (15/127) and America (13/127); there was no reported country of origin for 2 datasets and 3 datasets contained patients from multiple continents; the latter were multi-center cohorts. A total of 16,754 different patients were enrolled within these studies (avoiding double-counting samples that had been used for two or more analyses). Median sample size was 90 (IQR, 82; range, 30–997) and 69/127 datasets contained less than 100 participants (50 of which datasets came from China).

#### Mapping of lncRNA prognostic data

**Fig. 2:**
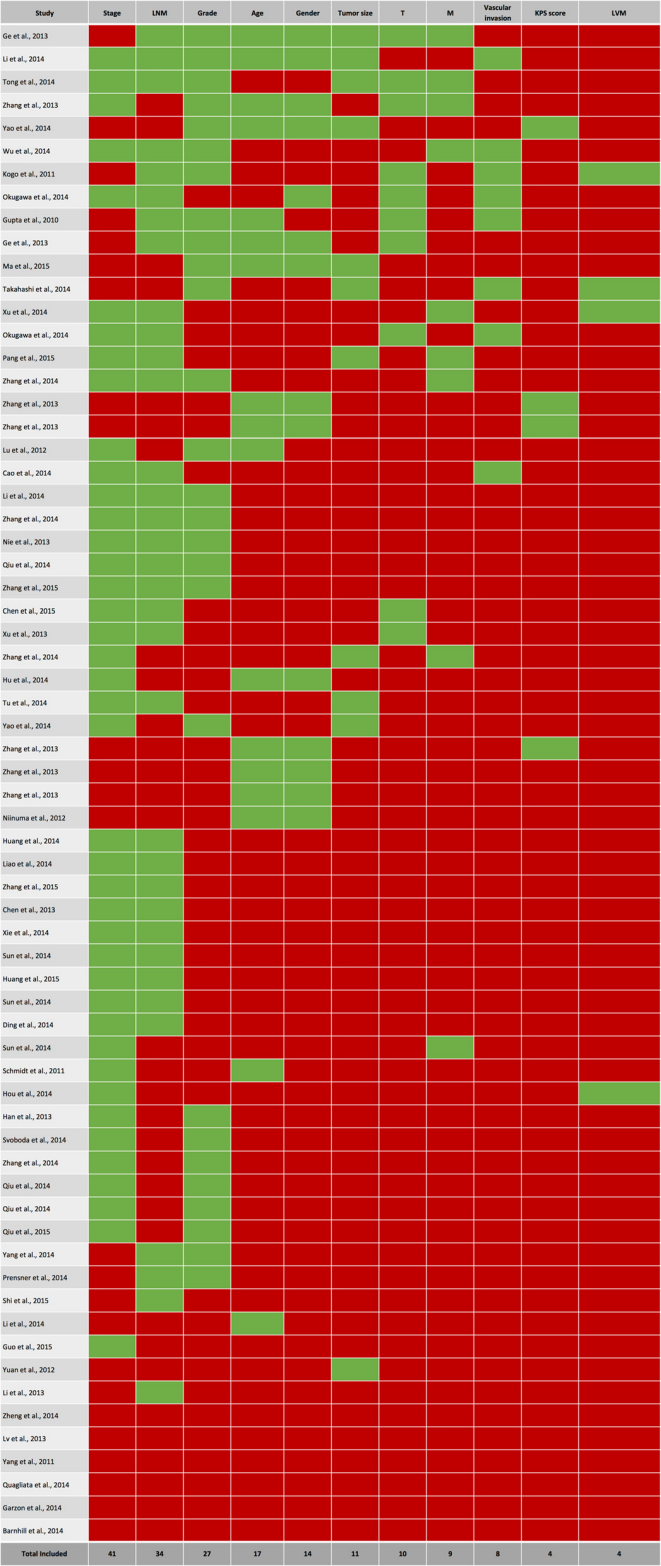
The covariates used within the multivariable models fitted by each paper. This is a data microarray in which the studies run along the Y-axis and the covariates run along the X-axis. Only the factors used three or more times are shown in this figure for convenience; refer to Additional file 3: Figure S1 for a data microarray illustrating all covariates studied. Rows and columns are ordered in descending order, based on how many times each covariate was included in the multivariable models fitted by each study. Where patterns were similar between studies or covariates, those papers or covariates were placed next to each other. It is evident that very few studies included the same covariates within their models and that less than half of the studies included both Stage and Grade within those models. Interestingly, according to Additional file 3: Figure S1, the majority of studies included at least one covariate within their model that had not been included in any other study. Green = Included in the multivariable model; Red = Not included in the multivariable model. LNM = Lymph node metastasis; T = Depth of invasion; M = Metastasis; KPS score = Karnofsky Performance Status score (a measure of functional impairment); LVM = Lymphovascular metastasis

The eligible reports studied 18 types of cancer, top three most studied of which were gastric cancer (*n* = 16 datasets), lung cancer (*n* = 15) and colorectal cancer (*n* = 15) (Table 1). Almost half of the reports studied cancer related to the gastrointestinal tract (57/127 datasets). OS was assessed in 92/111 studies (83 %), RFS in 36 (32 %), DSS in 10 (9 %), MFS in 9 (8 %) and PFS in 6 (5 %). The majority of studies did not appear to choose what lncRNAs to study on the basis of agnostic reports (77 %, 85/111). For 98/127 datasets (77 %), there was no information regarding adjuvant treatment; for the 29 studies providing information regarding treatment, only 4 datasets indicated that their patients were treated homogeneously. In addition to survival analysis, 68 % (76/111) of the identified studies attempted to further study their chosen lncRNAs *in vitro*, to corroborate the results of their survival analyses with mechanistic insights into the function of their chosen lncRNAs. Across 66 studies reporting multivariable analyses, 42 adjusted for stage of cancer (or all three components of the TNM staging) and 27 for grade of cancer; only 19/66 studies adjusted for both. Figure 2 displays a microarray of the covariates that have been studied more than three times within multivariable analyses (Additional file 3: Figure S1 displays the complete data microarray). Out of all 66 studies, 20 (30 %) studies adjusted for the same factors as at least one other paper and the most commonly encountered combination of factors adjusted for was Stage and Lymph Node Metastasis, which was seen in 6/66 studies. The median number of adjustment combinations matching between at least two papers was 1 (IQR, 0).

**Table 1:** Descriptive statistics of eligible studies

Characteristic	Subgroups	Frequency (%)
Year	2000–2013	13 (12 %)
	2013–2014	23 (21 %)
	2014–2014	0 (0 %)
	2014–2015	75 (68 %)
	N	111 (100 %)
Cancer site	Gastric	16 (13 %)
	Colorectal	15 (12 %)
	Lung	15 (12 %)
	Brain	12 (9 %)
	Esophageal	10 (8 %)
	Prostate	10 (8 %)
	Hepatic	9 (7 %)
	Breast	6 (5 %)
	Pancreatic	6 (5 %)
	Cervical	5 (4 %)
	Head and neck	4 (3 %)
	Ovarian	4 (3 %)
	Renal	4 (3 %)
	Urinary bladder	4 (3 %)
	Any	2 (2 %)
	Hematologic	2 (2 %)
	Endometrial	1 (1 %)
	GIST	1 (1 %)
	Neuroblastoma	1 (1 %)
	N	127 (100 %)
Agnostic ^a^	No	85 (77 %)
	Yes	21 (19 %)
	Agnostic parent	5 (5 %)
	N	111 (100 %)
Survival analysis	OS	92 (83 %)
	RFS	36 (32 %)
	DSS	10 (9 %)
	MFS	9 (8 %)
	PFS	6 (5 %)
	N	111 (100 %)
Quantification method	qRT-PCR	84 (66 %)
	ISH	28 (22 %)
	qPCR	11 (9 %)
	qRT-PCR or ISH	2 (2 %)
	RT-qPCR	1 (1 %)
	Unreported	1 (1 %)
	N	127 (100 %)
Continent	Asia	94 (74 %)
	Europe	15 (12 %)
	America	13 (10 %)
	Multiple	3 (2 %)
	Unreported	2 (2 %)
	N	127 (100 %)
Study design	Unreported	106 (83 %)
	Retrospective	19 (15 %)
	Prospective	2 (2 %)
	N	127 (100 %)
Sampling method	Unreported	113 (89 %)
	Consecutive	5 (4 %)
	Population	5 (4 %)
	Random	4 (3 %)
	N	127 (100 %)
Tissue preservation ^b^	L	66 (52 %)
	Unreported	34 (27 %)
	P	18 (14 %)
	LP	6 (5 %)
	L + RNALater	3 (2 %)
	N	127 (100 %)
Pre-biopsy treatment	No	77 (61 %)
	Unreported	46 (36 %)
	Yes	4 (3 %)
	N	127 (100 %)
Post-biopsy treatment	Unreported	98 (77 %)
	Yes	27 (21 %)
	No	2 (2 %)
	N	127 (100 %)
Total number of lncRNAs studied	1	87 (78 %)
	2–10	4 (4 %)
	11–45033	15 (14 %)
	Unreported	5 (5 %)
	N	111 (100 %)
Outcomes	Clinical and Non-clinical	76 (68 %)
	Clinical only	35 (32 %)
	N	111 (100 %)
Use of validation method for survival	Unreported	99 (89 %)
	External	5 (5 %)
	Internal	4 (4 %)
	Both	2 (2 %)
	Yes	1 (1 %)
	N	111 (100 %)

These data are based on 111 studies of 127 datasets. N refers to the total number of observations for each characteristic

^a^ Agnostic studies are those in which no prior knowledge is assumed regarding the choice of lncRNA to be studied

^b^ Tissue preservation: L (liquid nitrogen), P (paraffin-embedded), LP (liquid nitrogen and/or paraffin-embedded

#### Overall survival

Out of 92 studies reporting on OS, 87 studies (representing 111/127 analyses, as explained in Additional file 4: Table S1) provided effect estimates, out of which two were completely excluded due to reporting inconsistent effect sizes, as indicated in the Methods [35, 37]. The 85 remaining studies provided effect estimates on 53 lncRNAs and 6 multi-lncRNA risk score scales. The three most frequently studied lncRNAs within OS analyses were HOTAIR (*n* = 29 effect estimates), MALAT1 (*n* = 8) and GAS 5, H19 and PVT1 (*n* = 4 for each). Most individual lncRNAs (42/53) were only studied once (Table 2). Only 7 lncRNAs were studied at least three times in association to OS and for 6 of them more than half of the studies showed statistically significant *p*-values. These lncRNAs were studied in the context of a median of 4 different types of cancer (IQR, 3). Out of the 52 individual or groups of lncRNAs studied less than three times, 44 were always reported significantly associated to OS. Overall, of the 92 studies reporting on OS (but not necessarily quoting an effect estimate), 88 (96 %) reported at least one statistically significant result for association with prognosis.

**Table 2:** Details of the lncRNAs studied

LncRNA	Times studied	Number of cancer types (sample size)	Median (IQR)	Times significant (%)
HOTAIR	29	13 (3886)	100 (69)	28 (97 %)
MALAT1	8	7 (1135)	136 (53)	5 (62 %)
H19	4	2 (440)	77 (57)	2 (50 %)
PVT1	4	4 (420)	87 (24)	4 (100 %)
GAS5	4	4 (369)	96 (19)	4 (100 %)
SChLAP1	3	1 (1396)	357 (440)	3 (100 %)
6 lncRNA risk score	3	1 (281)	42 (94)	1 (33 %)
CCAT2	2	2 (1226)	613 (384)	1 (50 %)
LncR1 vs LncR2 vs LncR3	2	1 (759)	380 (96)	2 (100 %)
ENSG00000261582	2	2 (576)	288 (199)	2 (100 %)
MVIH	2	2 (257)	128 (86)	2 (100 %)
LOC285194	2	2 (227)	114 (28)	2 (100 %)
PCAT1	2	2 (212)	106 (2)	2 (100 %)
SPRY4-IT1	2	2 (190)	95 (3)	2 (100 %)
UCA1	2	2 (170)	85 (5)	2 (100 %)
GHET1	2	2 (122)	61 (19)	2 (100 %)
MEG3	2	2 (116)	58 (14)	2 (100 %)

The following lncRNAs were studied once and found statistically significant: LINC00968, LINC01234, LINC00476, FLG-AS1, HOTTIP, TC0101686, TC0100223. The following lncRNAs were studied once and were not found significant: linc-UBC1, KIAA0495, PART1, MGC21881, MIAT, PAR5, ADAMTS9-AS2, BCAR4, XLOC_010588, FOXCUT, 3 lncRNA risk score, FENDRR, HIF1A-AS2, ANRIL, GAPLINC, MRUL, HEIH, HOXA13, 48 lncRNA risk score, BANCR, ZXF1, CARLo-5, GAS6-AS1, Sox2ot, TUG1, NAG7 - LINC00312, CAI2, TC0101441, ENST00000480739, BC008363, 80-gene SChLAP1 signature risk score, 167-gene SChLAP1 signature risk score, CADM1-AS1, RCCRT1, CCAT1. Significance in the table refers to *p*-value < 0.05, as this is what had been used by these studies

The ‘Times studied’ column refers to how many studies investigated each lncRNA. The ‘Number of cancer types’ column indicates in how many different cancer types each lncRNA was studied, with the total number of participants used to study each lncRNA in brackets. The ‘Median’ column indicates the median sample size for each cohort used to study each lncRNA, with the interquartile range (IQR) in brackets. The last column indicates how many times each lncRNA was found to be statistically significantly associated to prognosis and in brackets the relation of how many times it was found to be significant versus how many times it was studied

#### Meta-analysis for overall survival

**Fig. 3:**
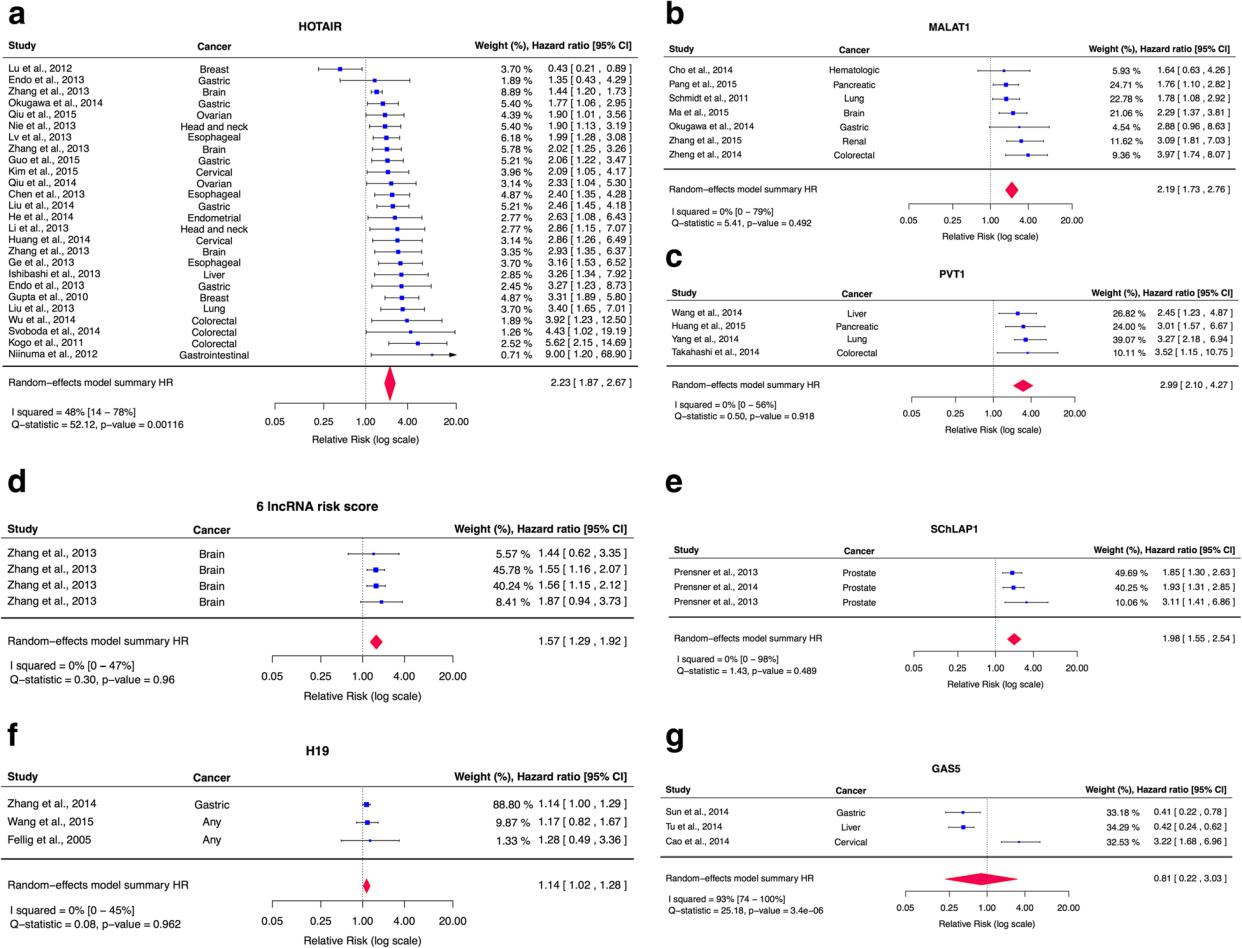
Forest plots for OS meta-analyses. We hereby illustrate the lncRNAs for which three or more studies reported OS. Each panel a-g corresponds to the meta-analysis of a different lncRNA: (**a**) HOTAIR, (**b**) MALAT1, (**c**) PVT1, (**d**) 6 lncRNA risk score, (**e**) SChLAP1, (**f**) H19 and (**g**) GAS5. The effect size for the estimate of each study is presented as a blue square proportional in size to the weight of that study. The confidence interval around that effect size is presented as a horizontal line. Where the confidence interval exceeds the range of our plot, an arrow has been placed. The vertical line across these estimates represents HR = 1 and any horizontal line crossing this vertical line represents a non-statistically significant result. The summary effect size is presented as a rhombus, the center of which represents the summary effect size and the width of which represents its confidence interval. It is evident that almost all studies quoted statistically significant results and that according to the available data, all meta-analyzed lncRNAs, apart from GAS5 (panel g), are statistically significantly associated to prognosis of OS in cancer. However, high between-study heterogeneity (based on the range of I^2^ estimates) indicates that these summary effect sizes are unreliable

**Fig. 4:**
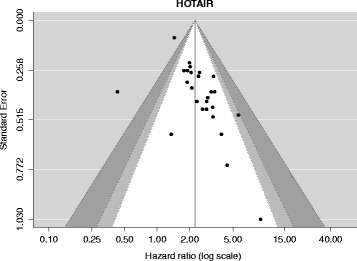
Funnel plot for the OS meta-analysis of HOTAIR. The meta-analysis for HOTAIR was analyzed with a funnel plot because it exceeded the pre-requisite of 10 studies. The Y-axis represents the Standard Error (SE), which serves as a measure of precision, where the higher the SE, the less precise the study. The HR has been plotted along the X-axis. The black dots map the effect size of HOTAIR on OS as this has been identified by each study. The light grey and dark grey areas respectively denote the 95 % and 99 % CI around the summary effect size. According to this plot, it is clear that the least precise studies tend to overestimate the effect size of HOTAIR on OS, skewing the summary effect size to the right (i.e. leading to a more strongly positive summary effect size)

A meta-analysis of OS was done for all 7 individual or groups of lncRNAs having been studied three or more times (Fig. 3; Table 3; Additional file 1: Table S2). For *p*-value < 0.0005, 5 lncRNAs were statistically significantly associated to OS in all of our meta-analyses (HOTAIR, MALAT1, 6 lncRNA risk score, PVT1, SChLAP1) and 6/7 were statistically significant in all of our meta-analyses at *p*-value < 0.05 (H19; Additional file 1: Table S2). An increase in cellular expression of these lncRNAs was statistically significantly associated to a decrease in overall survival; GAS5 was not statistically significantly associated to OS in our meta-analyses. The funnel plot for HOTAIR (Fig. 4), which is the only lncRNA studied 10 or more times, indicates significant small-study effects (*p*-value = 0.0006), and this may be suggestive of publication bias. The summary effect size for HOTAIR also displays a moderate amount of between-study heterogeneity (I^2^, 48 %; 95 % CI, 14–78 %). The summary effects for the effect of HOTAIR on OS in cancers for which it was studied 3 or more times were: colorectal cancer (HR, 4.76; 95 % CI, 2.46–9.21), esophageal cancer (HR, 2.29; 95 % CI, 1.68–3.12) and glioma (HR, 1.71; 95 % CI, 1.25–2.34).

**Table 3:** The results of our meta-analysis for each lncRNA using ‘primarily multivariable’ data

LncRNA	Studies	HR (95 % CI)	*P*-value	I^2^ (95 % CI)	Observed (Expected, *p*-value)
HOTAIR	26	2.22 (1.86–2.65)	0.0000	49 % (14–79 %)	25 (18.2, *p*-value = 0.002)
MALAT1	7	2.03 (1.64–2.52)	0.0000	0 % (0–85 %)	5 (4.1, *p*-value = 0.707)
6 lncRNA risk score	4	1.57 (1.29–1.92)	0.0000	0 % (0–47 %)	2 (2.1, *p*-value = 1.000)
GAS5	4	0.81 (0.33–2.00)	0.6479	94 % (80–100 %)	4 (2.1, *p*-value = 0.128)
H19	4	1.16 (1.04–1.29)	0.0100	0 % (0–98 %)	2 (0.8, *p*-value = 0.170)
PVT1	4	2.99 (2.10–4.27)	0.0000	0 % (0–56 %)	4 (2.7, *p*-value = 0.309)
SChLAP1	3	1.98 (1.55–2.54)	0.0000	0 % (0–98 %)	3 (2.9, *p*-value = 1.000)

‘Studies’ refers to the number of studies included in the meta-analysis of each lncRNA. HR = Hazard Ratio, 95 % CI = 95 % Confidence Interval. I^2^ is a measure of between-study heterogeneity. The last column refers to how many statistically significant results had been reported by the included studies (Observed, O), how many were expected to be reported on the basis of each study’s power (Expected, E) and whether O and E are statistically significantly different from each other for each meta-analysis (*p*-value). Please refer to Additional file 1: Table S2 for a table illustrating all meta-analyses done (not only the one for primarily multivariable data) with all of the measures calculated

#### Other meta-analyses

The only type of survival analysis other than OS studied 3 or more times in relation to a specific lncRNA was MFS for HOTAIR. This was investigated within 4 different studies in relation to 4 different cancers (breast, colorectal, esophageal, head and neck). Meta-analysis of these studies identified a summary HR of 2.54 (95 % CI, 1.62–3.98) with no statistically significant heterogeneity (Q-statistic, 5.16; *p*-value = 0.16).

#### Heterogeneity metrics and excess significance

Statistically significant heterogeneity was only observed in HOTAIR analyses, but substantial estimates of I^2^ were common. For HOTAIR and OS, a sensitivity analysis excluding the only study reporting an inverse correlation of HOTAIR to cancer survival [33] generated a HR of 2.30 (95 % CI, 1.97-2.70) with I^2^ = 0 % (95 % CI, 0–59 %); for all other meta-analyses, no single study produced a major change in the I^2^.

There was excess significance across the whole field for overall survival and the binomial distribution revealed a two-tailed *p*-value of 0.0003, with O = 42 statistically significant results and E = 30 expected statistically significant results across all meta-analyses with 3 or more studies each on OS. As far as excess significance within lncRNAs studied 5 or more times is concerned, there was significant excess significance documented for HOTAIR (*p*-value = 0.002), but not MALAT1 (*p*-value = 0.46).

## Discussion

In this systematic review and meta-analysis we have tried to gather all published papers evaluating the prognostic ability of lncRNAs in cancer. We have identified that a large number of lncRNAs have been evaluated within the context of cancer prognosis. Most of them have been evaluated only once in a published paper. Almost all of the published papers report that lncRNAs are statistically significant predictors of survival. There was often substantial heterogeneity between studies in the strength of the predictive effect. There was also strong evidence for small-study effects and for excess significance. This picture may be due to genuine differences across studies, such as different cancers and populations under study, and different adjustments made in multivariable models. However, it is also highly compatible with the presence of substantial publication bias and other selective reporting bias in this field resulting in exaggerated effects in mostly small studies (most of which coming from China) and in an implausibly high prevalence of nominally significant results.

It is well recognized that published literature on prognosis and the identification of prognostic markers is characterized by poor methodological quality, significant publication bias and wide heterogeneity in aspects of sample selection, such as pre/post-biopsy treatment or tissue preservation methods, and analysis, such as multivariable modelling and determination of cutoff values [30, 46]. As such, meta-analyses of prognostic studies may elicit summary effect sizes that are unrealistic [47]. An evaluation of studies investigating the association of TP53 to risk of death by head and neck squamous cell carcinoma, identified that even though readily available effect sizes would confirm that TP53 is a strongly significant prognostic factor, after standardizing definitions of TP53 status and outcomes across papers and retrieving non-readily available information, this association was completely abrogated [48]. These issues may also apply to the lncRNA literature. No two studies of our dataset were identical in all of lncRNA, cancer site, cut-off value and multivariable modelling, suggesting substantial room for selective reporting of analyses that could be done with very different models and definitions. Moreover, we suspect that publication bias may also be operating in the field.

Of particular interest is the excess significance we identified across the field (*p*-value = 0.0003). Despite the poor translation of cancer biomarkers into clinical practice [39, 49–51], out of 1575 studies on cancer biomarkers published in 2005, 95.8 % reported statistically significant results and only 1.3 % did not report any kind of statistically significant results [52]. Indeed, as we have shown, this pattern is also prominent in the lncRNA cancer prognosis literature.

One way of reducing the selective reporting biases that have led to the above status quo and thus reducing lack of translatability, is transparency. The need to improve transparency has been mentioned repeatedly [39, 53]. Guidelines have been proposed to improve the reporting of prognostic markers (REMARK) [39, 51], multivariable prediction models (TRIPOD) [54] and genetic risk prediction studies [55]. Wider adoption of these guidelines may increase transparency, but it is unknown whether it will suffice to markedly reduce selective reporting.

In our cohort of studies, the extent of unreported items in Table 1, did not inspire confidence in transparency and completeness of reporting practices. We also documented minimal use of validation (12/111 studies, 11 %), despite reports stressing the necessity and importance of validation in identifying true effect size for prognostic tools [56, 57]. Furthermore, more than half of the identified studies had a sample size of less than 100. Small studies are known, both theoretically and empirically, to be associated with inflated estimates of effect size [58], not as much due to their limited sample size, as for lower quality standards, publication bias and selective reporting [59], which is why they lead to so-called ‘small-study effects’. Even though these have mostly been studied within the context of randomized-controlled trials, where they have been associated with a larger average effect size and at least double the between-study heterogeneity found in larger studies [60], similar problems may occur also in prognostic study research [43]. The meta-analysis for HOTAIR, which is the most widely studied lncRNA in the context of cancer prognosis, clearly indicates that smaller studies tend to be less precise and report a higher effect size than larger studies. Inflated effects are common in biomarker studies [61], and this may apply also for the results of lncRNAs.

Another interesting point of note is the Chinese provenance of most papers in our collection of eligible studies (78/111, 70 %). In a previous analysis of genetic studies, it was shown that there is a vast Chinese literature, and that papers from China tend to utilize smaller sample sizes yet reach statistical significance far more commonly than other papers [62]. This was attributed to more prominent publication bias against null results or other kinds of selection bias in pursuit of statistically significant results. Discrepancies between the Chinese literature and the rest of the world were also found in published meta-analyses of genomic data [63]. Chinese meta-analyses (1) focused on the results of studies investigating individual candidate genes rather than the results of genome-wide association studies and (2) used nominal significance (i.e. *p*-value < 0.05) rather than genome-wide *p*-value thresholds to identify statistically significant results.

Although there has been an explosion in the amount of identified potential biomarkers due to high throughput methods, unlike traditional methods of identifying molecules directly relevant to a known cellular event [49], very few have made their way to clinical practice, due to lack of appropriate evidence [50, 64, 65]. An important aspect in ascribing usefulness to a novel biomarker is their ability to add further predictive value, over and above the one already possible using known prognostic factors. Unfortunately, in our sample, despite most multivariable analyses identifying lncRNAs as a statistically significant predictor, only about 30 % of the reported prognostic effects were adjusted for the two classically most relevant predictors of cancer prognosis (i.e. Stage and Grade).

### Limitations

Our analysis has several limitations. First, given that this report is only based on the results of a single database (PubMed), it is possible that relevant papers may have been missed. Second, our analysis utilized the Medical Subject Heading (MeSH) ‘Humans’ to limit our search results to those studies conducted in humans. Even though this is accepted practice and has been used previously in similar studies [5], that label is added to papers at the point of indexing, and thus some papers that were published close to our search date (September 26, 2015) and had not been MeSH-labeled yet, would have been missed. We performed an updated search (June 5, 2016) for papers that did not have a Human [MeSH] and had been published before 2015 and found only two small studies [66, 67] that could potentially qualify for inclusion for the outcome of survival. This is a field with prolific literature and a substantial number of papers have continued to appear after our September 2015 search and will probably continue to appear in the near future. Third, our meta-analysis has attempted to combine multiple studies that are known to be heterogeneous in terms of cancer site and provenance of patient populations. Our estimates of heterogeneity metrics have wide 95 % confidence intervals [42]. Fourth, on 51 occasions we had to calculate HRs ourselves based on data provided within the papers, which may not have provided the most accurate estimate of the HR possible, as most of the time these data were extracted from Kaplan-Meier curves. However, this practice has not been shown to yield results significantly different from direct methods of HR estimation [29]. Fifth, even though every effort was made to exclude analyses of the same lncRNA using the same dataset of patients, it is possible that some overlapping data have been included, if their authors have made no hint as to the presence of overlap.

## Conclusions

In conclusion, we have gathered a substantial amount of prognostic data regarding the association of various lncRNAs and survival. Our analysis identified a significant number of studies, most of which have been published within the last 2 years and most of which are of small sample size. Even though our systematic review and meta-analyses identified that almost all lncRNAs identified are statistically significant predictors of OS, it is very difficult to know the importance of these associations, given the detection of excess significance, small-study effects and the known difficulties with analyzing prognostic studies. Larger studies, ideally with collaborative teams using standardized approaches to measurement, adjustment, analysis, and reporting, will offer better insights into the prognostic value of lncRNAs.

## Abbreviations

RNA, Ribonucleic acid; ncRNAs, Noncoding RNAs; LncRNAs, Long noncoding RNAs; LincRNA, large intergenic non-coding RNAs; T-UCRs, transcribed ultraconserved regions; miR, microRNA; HOTAIR (HOmeobox (HOX) Transcript AntIsense RNA); PRC2, Polycomb Repressive Complex 2; HR, Hazard Ratio; CI, Confidence Interval; IQR, Interquartile range; DSS, Disease-specific survival; MFS, Metastasis-free survival, OS, Overall/cumulative survival; PFS, Progression/event/disease-free survival; RFS, Recurrence-free survival; O, Number of observed events; E, Number of expected events; PCR, Polymerase chain reaction; qPCR, Quantitative PCR; qRT-PCR, Quantitative real-time PCR; RT-qPCR, real-time quantitative PCR; ISH, *in situ* hybridization; LNM, Lymph node metastasis; LVM, Lymphovascular metastasis

## Additional files

Additional file 1: Table S2. A table presenting all meta-analyses done. 'Analysis' refers to the the type of data used in each meta-analysis, as this was explained in Methods. 'Studies' refers to the number of studies included within each meta-analysis. Columns 'HR' and '95 % CI' refer to the summary Hazard Ratio (HR) of each meta-analysis with its 95 % Confidence Interval (CI) (lower and upper limit). 'Tau' refers to the squared root of the estimate of between-study variance in each of our random-effects meta-analyses. Columns 'I and '95 % CI' refer to a measure of between-study heterogeneity and its corresponding 95 % CI. 'Q-statistic' and its 'P-value' refer to Cochran's Q measure of heterogeneity with its p-value. 'Observed', 'Expected' and 'P-value (binomial)' refer to the observed and expected amount of statistically significant results and the comparison between the two, as this was described in Methods. (XLSX 50.7 kb) Additional file 2: The studies eligible for systematic review. (DOC 444 kb) Additional file 3: Figure S1. The covariates included within the multivariable models fitted by each paper. This is a data microarray in which the studies run along the Y-axis and the covariates run along the X-axis. Rows and columns are ordered in descending order, based on the total number each covariate was included in the multivariable models fitted by each study. Where patterns were similar between studies or covariates, those studies or covariates were placed next to each other. (PDF 82 kb) Additional file 4: Table S1. Explanation of how 92 studies provided data regarding 127 analyses. (DOC 36 kb)
