# Supplementary material for: Long noncoding RNAs as novel predictors of survival in human cancer: a systematic review and meta-analysis
Source: Mol Cancer. 2016 Jun 28;15:50. doi: 10.1186/s12943-016-0535-1 (PMC4924330; doi:10.1186/s12943-016-0535-1)
Supplement: Additional file 2: — The studies eligible for systematic review. (DOC 444 kb) [file 12943_2016_535_MOESM2_ESM.doc]

**Table 1.** The studies eligible for systematic review.

| **Study** | **Cancer site** | **lncRNAs** | **N** | **Quantification method** | **Tissue preservation** | **Pre-biopsy treatment** | **Post-biopsy treatment** | **Survival** |
| --- | --- | --- | --- | --- | --- | --- | --- | --- |
| Gupta et al., 2010 [A1] | Breast | HOTAIR | 132 | qRT-PCR | LP | N | Y; Y; | MFS, OS |
| Wang et al., 2015 [A2] | Any | Multiple | 248 | Unreported | U | U | U | OS |
| Li et al., 2014 [A3] | Urinary bladder | GHET1 | 80 | qRT-PCR | U | N | U | OS |
| Ariel et al., 2000 [A4] | Urinary bladder | H19 | 48 | ISH | P | U | U | RFS |
| Yan et al., 2014 [A5] | Urinary bladder | HOTAIR | 110 | qRT-PCR | LP | U | Y | RFS, OS |
| He et al., 2013 [A6] | Urinary bladder | linc-UBC1 | 102 | qRT-PCR | L | U | U | MFS, OS |
| Zhang et al., 2013 [A7] | Brain | Multiple | 213; 68; 101 | qRT-PCR | U | U | Y | OS |
| Yao et al., 2014 [A8] | Brain | ADAMTS9-AS2 | 70 | qRT-PCR | L | N | Y | OS |
| Ma et al., 2015 [A9] | Brain | MALAT1 | 118 | qRT-PCR | L | N | U | OS |
| Li et al., 2014 [A10] | Brain | Multiple | 475; 284 | ISH | L | U | Y | OS |
| Godinho et al., 2010 [A11] | Breast | BCAR4 | 280 | qRT-PCR | U | Y | Y | PFS, MFS, RFS, OS |
| Redis et al., 2013 [A12] | Breast | CCAT2 | 997; 56 | qRT-PCR | L | N | Y; Y | MFS, OS |
| Lu et al., 2012 [A13] | Breast | HOTAIR | 348 | qRT-PCR | L | U | Y | RFS, OS |
| Sorensen et al., 2013 [A14] | Breast | HOTAIR | 164 | ISH | U | N | N | MFS |
| Cao et al., 2014 [A15] | Cervical | GAS5 | 102 | qRT-PCR | L | N | U | OS |
| Li et al., 2015 [A16] | Cervical | HOTAIR | 118 | qRT-PCR | U | U | U | RFS, OS |
| Kim et al., 2015 [A17] | Cervical | HOTAIR | 111 | qRT-PCR | L | N | U | OS |
| Huang et al., 2014 [A18] | Cervical | HOTAIR | 218 | qRT-PCR | L | N | U | RFS, OS |
| Liao et al., 2014 [A19] | Cervical | XLOC_010588 | 218 | qRT-PCR | L | N | U | RFS, OS |
| Zheng et al., 2014 [A20] | Colorectal | MALAT1 | 146 | qRT-PCR | LR | N | U | RFS, OS |
| He et al., 2014 [A21] | Colorectal | CCAT1 | 48 | qPCR | L | N | U | OS |
| Takahashi et al., 2014 [A22] | Colorectal | PVT1 | 164 | qRT-PCR | LR | N | U | OS |
| Svoboda et al., 2014 [A23] | Colorectal | HOTAIR | 73 | qRT-PCR | U | N | Y | OS |
| Wu et al., 2014 [A24] | Colorectal | HOTAIR | 120 | qPCR | L | N | Y | MFS, OS |
| Iguchi et al., 2015 [A25] | Colorectal | ATB | 124 | qRT-PCR | U | U | U | RFS |
| Kogo et al., 2011 [A26] | Colorectal | HOTAIR | 100 | qRT-PCR | L | N | U | OS |
| Qi et al., 2013 [A27] | Colorectal | LOC285194 | 82 | qRT-PCR | L | N | U | RFS, DSS |
| Ge et al., 2013 [A28] | Colorectal | PCAT1 | 108 | qRT-PCR | L | N | U | OS |
| Han et al., 2014 [A29] | Colorectal | UCA1 | 80 | RT-qPCR | U | U | U | OS |
| Hu et al., 2014 [A30] | Colorectal | Multiple | 436; 177 | qRT-PCR; ISH | L | N | Y | RFS, DSS |
| Chen et al., 2014 [A31] | Colorectal | Multiple | 359 | ISH | L | N | U | PFS, RFS |
| Shi et al., 2014 [A32] | Colorectal | RP11-462C24.1 | 86 | qRT-PCR | L | N | U | DSS |
| He et al., 2014 [A33] | Endometrial | HOTAIR | 145 | ISH | L | N | U | OS |
| Zhang et al., 2015 [A34] | Esophageal | CCAT2 | 229 | qRT-PCR | L | N | U | OS |
| Tong et al., 2014 [A35] | Esophageal | LOC285194 | 142 | qRT-PCR | L | Y | U | RFS, OS |
| Pan et al., 2014 [A36] | Esophageal | FOXCUT | 82 | ISH | L | N | U | OS |
| Ge et al., 2013 [A37] | Esophageal | HOTAIR | 137 | qPCR | LP | N | U | MFS, OS |
| Lv et al., 2013 [A38] | Esophageal | HOTAIR | 93 | qRT-PCR; ISH; | P | N | U | OS |
| Chen et al., 2013 [A39] | Esophageal | HOTAIR | 78 | qRT-PCR | L | N | U | OS |
| Shi et al., 2015 [A40] | Esophageal | PCAT1 | 104 | qRT-PCR | L | N | U | OS |
| Xie et al., 2014 [A41] | Esophageal | SPRY4-IT1 | 92 | qRT-PCR | L | N | U | OS |
| Li et al., 2014 [A42] | Esophageal | UCA1 | 90 | qRT-PCR | L | N | U | OS |
| Li et al., 2014 [A43] | Esophageal | Multiple | 119 | ISH | U | U | U | OS |
| Xu et al., 2014 [A44] | Gastric | FENDRR | 158 | qRT-PCR | U | U | U | RFS, OS |
| Sun et al., 2014 [A45] | Gastric | GAS5 | 89 | qRT-PCR | L | U | U | RFS, OS |
| Zhang et al., 2014 [A46] | Gastric | H19 | 80 | qPCR | U | N | U | OS |
| Chen et al., 2015 [A47] | Gastric | HIF1A-AS2 | 83 | qPCR | U | N | U | OS |
| Guo et al., 2015 [A48] | Gastric | HOTAIR | 515 | qRT-PCR | L | N | U | OS |
| Endo et al., 2013 [A49] | Gastric | HOTAIR | 68 | qRT-PCR | LP | N | U | OS |
| Sun et al., 2014 [A50] | Gastric | MEG3 | 72 | qRT-PCR | L | N | U | OS |
| Xu et al., 2013 [A51] | Gastric | HOTAIR | 83 | qRT-PCR | L | U | U | OS |
| Okugawa et al., 2014 [A52] | Gastric | MALAT1; HOTAIR; | 150 | qRT-PCR | U | N | U | OS |
| Zhang et al., 2014 [A53] | Gastric | ANRIL | 120 | qRT-PCR | U | U | U | RFS, OS |
| Hu et al., 2014 [A54] | Gastric | GAPLINC | 90 | ISH | U | U | U | OS |
| Yang et al., 2014 [A55] | Gastric | GHET1 | 42 | qRT-PCR | U | U | U | OS |
| Li et al., 2014 [A56] | Gastric | H19 | 74 | qRT-PCR | LR | U | U | OS |
| Liu et al., 2014 [A57] | Gastric | HOTAIR | 78 | qRT-PCR | LP | N | U | OS |
| Wang et al., 2014 [A58] | Gastric | MRUL | 40 | qRT-PCR; ISH | U | Y | U | OS |
| Xu et al., 2014 [A59] | Gastric; Colorectal | LSINCT5 | 71; 74 | qPCR | U | N | U | RFS, DSS |
| Niinuma et al., 2012 [A60] | GIST | HOTAIR | 39 | qRT-PCR | L | U | U | OS |
| Zhang et al., 2013 [A61] | Brain | HOTAIR | 220; 75; 475; 64 | ISH; ISH; ISH; qRT-PCR | P; P; L; L | N; N; U; U | Y | OS |
| Cho et al., 2014 [A62] | Hematologic | MALAT1 | 45 | qRT-PCR | L | N | Y | PFS, OS |
| Esteves et al., 2005 [A63] | Head and neck | H19 | 41 | qRT-PCR | U | N | U | DSS |
| Tu et al., 2014 [A64] | Liver | GAS5 | 71 | qRT-PCR | P | N | U | RFS, OS |
| Zhang et al., 2013 [A65] | Hepatic | H19 | 113 | qRT-PCR | P | U | U | DSS |
| Ishibashi et al., 2013 [A66] | Liver | HOTAIR | 64 | qRT-PCR | L | U | U | RFS, OS |
| Yang et al., 2011 [A67] | Liver | HEIH | 85 | qRT-PCR | U | U | U | OS |
| Quagliata et al., 2014 [A68] | Liver | HOTTIP; HOXA13; | 52 | qRT-PCR | L | N | U | OS |
| Yang et al., 2011 [A69] | Liver | HOTAIR | 60 | qRT-PCR | U | U | U | RFS |
| Lai et al., 2012 [A70] | Liver | MALAT1 | 52 | qRT-PCR | L | U | U | RFS |
| Yuan et al., 2012 [A71] | Liver | MVIH | 215 | qRT-PCR | L | N | U | RFS, OS |
| Wang et al., 2014 [A72] | Liver | PVT1 | 89 | qRT-PCR | P | U | U | RFS, OS |
| Fellig et al., 2005 [A73] | Any | H19 | 38 | ISH | P | U | U | OS |
| Li et al., 2013 [A74] | Head and neck | HOTAIR | 72 | qPCR | L | N | U | OS |
| Garzon et al., 2014 [A75] | Hematologic | 48-lncRNA score | 148 | ISH | L | N | Y | RFS, OS |
| Sun et al., 2014 [A76] | Lung | BANCR | 113 | qPCR | L | N | Y | PFS, RFS, OS |
| Zhang et al., 2014 [A77] | Lung | ZXF1 | 62 | qRT-PCR | U | U | U | OS |
| Luo et al., 2014 [A78] | Lung | CARLo-5 | 62 | qRT-PCR | L | N | U | OS |
| Han et al., 2013 [A79] | Lung | GAS6-AS1 | 50 | qRT-PCR | L | N | U | OS |
| Nakagawa et al., 2013 [A80] | Lung | HOTAIR | 77 | qRT-PCR | L | N | U | RFS |
| Liu et al., 2013 [A81] | Lung | HOTAIR | 42 | qRT-PCR | L | N | U | OS |
| Schmidt et al., 2011 [A82] | Lung | MALAT1 | 222 | ISH | P | U | U | OS |
| Lu et al., 2013 [A83] | Lung | MEG3 | 44 | qRT-PCR | L | U | U | OS |
| Nie et al., 2014 [A84] | Lung | MVIH | 42 | qPCR | L | N | U | OS |
| Yang et al., 2014 [A85] | Lung | PVT1 | 82 | qRT-PCR | L | N | U | OS |
| Hou et al., 2014 [A86] | Lung | Sox2ot | 83 | qRT-PCR | L | U | U | OS |
| Zhang et al., 2014 [A87] | Lung | TUG1 | 192 | qRT-PCR | U | N | U | OS |
| Shen et al., 2015 [A88] | Lung | MALAT1 | 78 | qRT-PCR | U | U | U | RFS |
| Ono et al., 2014 [A89] | Lung | HOTAIR | 35 | qRT-PCR | L | Y | Y | RFS, DSS |
| Du et al., 2013 [A90] | Brain; Ovarian; Lung; Prostate; | Multiple | 220; 487; 89; 150 | ISH | U; L; L; L | U; N; N; U | U | PFS, OS |
| Zhang et al., 2013 [A91] | Head and neck | NAG7 (LINC00312) | 96 | ISH | LP | U | U | RFS, OS |
| Nie et al., 2013 [A92] | Head and neck | HOTAIR | 160 | ISH | P | N | U | MFS, RFS, OS |
| Barnhill et al., 2014 [A93] | Neuroblastoma | CAI2 | 62 | qRT-PCR | U | U | U | RFS, OS |
| Qiu et al., 2014 [A94] | Ovarian | Multiple | 95 | qRT-PCR | L | N | U | OS |
| Qiu et al., 2014 [A95] | Ovarian | HOTAIR | 64 | qRT-PCR | L | N | U | RFS, OS |
| Qiu et al., 2015 [A96] | Ovarian | HOTAIR | 68 | qRT-PCR | L | N | U | OS |
| Liu et al., 2014 [A97] | Pancreatic | MALAT1 | 45 | qRT-PCR | P | U | U | DSS |
| Huang et al., 2015 [A98] | Pancreatic | PVT1 | 85 | qRT-PCR | U | N | U | OS |
| Pang et al., 2015 [A99] | Pancreatic | MALAT1 | 126 | qRT-PCR | L | N | U | OS |
| Sun et al., 2014 [A100] | Pancreatic | ENST00000480739 | 35 | qRT-PCR | L | N | U | OS |
| Ding et al., 2014 [A101] | Pancreatic | LOC285194 | 85 | qRT-PCR | U | N | U | OS |
| Li et al., 2014 [A102] | Pancreatic | Multiple | 30 | qRT-PCR | P | U | U | OS |
| Prensner et al., 2014 [A103] | Prostate | PCGEM1; PRNCR1; SChLAP1 | 235 | ISH | P | N | Y | DSS |
| Mehra et al., 2014 [A104] | Prostate | SChLAP1 | 160 | ISH | U | U | U | RFS |
| Malik et al., 2014 [A105] | Prostate | PCAT29 | 51 | qPCR | U | U | U | RFS |
| Prensner et al., 2013 [A106] | Prostate | SChLAP1 | 357; 79; 235 | qRT-PCR; ISH; qRT-PCR | P; L; P | N; U; N | U | PFS, RFS, DSS, OS |
| Prensner et al., 2014 [A107] | Prostate | SChLAP1 | 545; 232; 183 | ISH | P | N | Y; Y; N | MFS, RFS, OS |
| Yao et al., 2014 [A108] | Renal | CADM1-AS1 | 64 | qRT-PCR | L | N | U | OS |
| Zhang et al., 2014 [A109] | Renal | SPRY4-IT1 | 98 | qRT-PCR | L | N | U | OS |
| Zhang et al., 2015 [A110] | Renal | MALAT1 | 106 | qRT-PCR | L | N | U | OS |
| Song et al., 2014 [A111] | Renal | RCCRT1 | 40 | qRT-PCR | L | U | U | OS |

Please note that these citations refer to those in Appendix 1 and that some papers have an identical citation in letter format. Where more than 3 lncRNAs were studied by a paper, instead of quoting all lncRNAs this was denoted as ‘Multiple’. The column ‘N’ refers to the sample size of each dataset utilized by the study. The ‘Quantification method’ refers to how the presence of lncRNAs was quantified; qRT-PCR = quantitative Real Time – PCR, ISH = *in situ* hybridization. For ‘Tissue preservation’: L = Liquid nitrogen, P = Paraffin-embedded, U = Unreported. For ‘Pre-biopsy treatment‘ and ‘Post-biopsy treatment’: Y = Yes, N = No, U = Unreported. For ‘Survival’: DSS = Disease-Specific Survival, MFS = Metastasis-Free Survival, OS = Overall/cumulative Survival, PFS = Progression/event/disease-Free Survival, RFS = Recurrence-Free Survival. In case more than one datasets were used, the sequence in which information appears within the row is specific to each dataset; for example, Prensner et al., 2013 [106] used 4 datasets, the first of which had a sample size of 357, was analyzed using qRT-PCR, preserved tissue by embedding it in paraffin and so on, the second of which had a sample size of 79, was analyzed using ISH, preserved tissue using Liquid Nitrogen and so on, for each dataset.

# Appendix 1

## References of eligible studies

1. Gupta RA, Wang KC, Hung T, West RB, Sukumar S, Chang HY. Long non-coding RNA HOTAIR reprograms chromatin state to promote cancer metastasis. Nature. 2010;464:1071–6.

2. Wang P, Ning S, Zhang Y, Li R, Ye J, Zhao Z, et al. Identification of lncRNA-associated competing triplets reveals global patterns and prognostic markers for cancer. Nucleic Acids Res. Oxford University Press; 2015;43:3478–89.

3. Li L-J, Zhu J-L, Bao W-S, Chen D-K, Huang W-W, Weng Z-L. Long noncoding RNA GHET1 promotes the development of bladder cancer. Int J Clin Exp Pathol. 2014;7:7196–205.

4. Ariel I, Sughayer M, Fellig Y, Pizov G, Ayesh S, Podeh D, et al. The imprinted H19 gene is a marker of early recurrence in human bladder carcinoma. Mol Pathol. 2000;53:320–3.

5. Yan B, Gu W, Yang Z, Gu Z, Yue X, Gu Q, et al. Downregulation of a long noncoding RNA-ncRuPAR contributes to tumor inhibition in colorectal cancer. Tumour Biol. Springer Netherlands; 2014;35:11329–35.

6. He W, Cai Q, Sun F, Zhong G, Wang P, Liu H, et al. linc-UBC1 physically associates with polycomb repressive complex 2 (PRC2) and acts as a negative prognostic factor for lymph node metastasis and survival in bladder cancer. Biochim Biophys Acta. 2013;1832:1528–37.

7. Zhang X-Q, Sun S, Lam K-F, Kiang KM-Y, Pu JK-S, Ho AS-W, et al. A long non-coding RNA signature in glioblastoma multiforme predicts survival. Neurobiol Dis. 2013;58:123–31.

8. Yao J, Zhou B, Zhang J, Geng P, Liu K, Zhu Y, et al. A new tumor suppressor LncRNA ADAMTS9-AS2 is regulated by DNMT1 and inhibits migration of glioma cells. Tumour Biol. Springer Netherlands; 2014;35:7935–44.

9. Ma K-X, Wang H-J, Li X-R, Li T, Su G, Yang P, et al. Long noncoding RNA MALAT1 associates with the malignant status and poor prognosis in glioma. Tumour Biol. Springer Netherlands; 2015;36:3355–9.

10. Li R, Qian J, Wang Y-Y, Zhang J-X, You Y-P. Long noncoding RNA profiles reveal three molecular subtypes in glioma. CNS Neurosci Ther. 2014;20:339–43.

11. Godinho MFE, Sieuwerts AM, Look MP, Meijer D, Foekens JA, Dorssers LCJ, et al. Relevance of BCAR4 in tamoxifen resistance and tumour aggressiveness of human breast cancer. Br J Cancer. 2010;103:1284–91.

12. Redis RS, Sieuwerts AM, Look MP, Tudoran O, Ivan C, Spizzo R, et al. CCAT2, a novel long non-coding RNA in breast cancer: expression study and clinical correlations. Oncotarget. 2013;4:1748–62.

13. Lu L, Zhu G, Zhang C, Deng Q, Katsaros D, Mayne ST, et al. Association of large noncoding RNA HOTAIR expression and its downstream intergenic CpG island methylation with survival in breast cancer. Breast Cancer Res Treat. Springer US; 2012;136:875–83.

14. Sorensen KP, Thomassen M, Tan Q, Bak M, Cold S, Burton M, et al. Long non-coding RNA HOTAIR is an independent prognostic marker of metastasis in estrogen receptor-positive primary breast cancer. Breast Cancer Res Treat. Springer US; 2013;142:529–36.

15. Cao S, Liu W, Li F, Zhao W, Qin C. Decreased expression of lncRNA GAS5 predicts a poor prognosis in cervical cancer. Int J Clin Exp Pathol. 2014;7:6776–83.

16. Li J, Wang Y, Yu J, Dong R, Qiu H. A high level of circulating HOTAIR is associated with progression and poor prognosis of cervical cancer. Tumour Biol. Springer Netherlands; 2015;36:1661–5.

17. Kim HJ, Lee DW, Yim GW, Nam EJ, Kim S, Kim SW, et al. Long non-coding RNA HOTAIR is associated with human cervical cancer progression. Int J Oncol. Spandidos Publications; 2015;46:521–30.

18. Huang L, Liao L-M, Liu A-W, Wu J-B, Cheng X-L, Lin J-X, et al. Overexpression of long noncoding RNA HOTAIR predicts a poor prognosis in patients with cervical cancer. Arch Gynecol Obstet. Springer Berlin Heidelberg; 2014;290:717–23.

19. Liao L-M, Sun X-Y, Liu A-W, Wu J-B, Cheng X-L, Lin J-X, et al. Low expression of long noncoding XLOC_010588 indicates a poor prognosis and promotes proliferation through upregulation of c-Myc in cervical cancer. Gynecol Oncol. 2014;133:616–23.

20. Zheng H-T, Shi D-B, Wang Y-W, Li X-X, Xu Y, Tripathi P, et al. High expression of lncRNA MALAT1 suggests a biomarker of poor prognosis in colorectal cancer. Int J Clin Exp Pathol. 2014;7:3174–81.

21. He X, Tan X, Wang X, Jin H, Liu L, Ma L, et al. C-Myc-activated long noncoding RNA CCAT1 promotes colon cancer cell proliferation and invasion. Tumour Biol. Springer Netherlands; 2014;35:12181–8.

22. Takahashi Y, Sawada G, Kurashige J, Uchi R, Matsumura T, Ueo H, et al. Amplification of PVT-1 is involved in poor prognosis via apoptosis inhibition in colorectal cancers. Br J Cancer. 2014;110:164–71.

23. Svoboda M, Slyskova J, Schneiderova M, Makovicky P, Bielik L, Levy M, et al. HOTAIR long non-coding RNA is a negative prognostic factor not only in primary tumors, but also in the blood of colorectal cancer patients. Carcinogenesis. Oxford University Press; 2014;35:1510–5.

24. Wu Z-H, Wang X-L, Tang H-M, Jiang T, Chen J, Lu S, et al. Long non-coding RNA HOTAIR is a powerful predictor of metastasis and poor prognosis and is associated with epithelial-mesenchymal transition in colon cancer. Oncol Rep. Spandidos Publications; 2014;32:395–402.

25. Iguchi T, Uchi R, Nambara S, Saito T, Komatsu H, Hirata H, et al. A long noncoding RNA, lncRNA-ATB, is involved in the progression and prognosis of colorectal cancer. Anticancer Res. 2015;35:1385–8.

26. Kogo R, Shimamura T, Mimori K, Kawahara K, Imoto S, Sudo T, et al. Long noncoding RNA HOTAIR regulates polycomb-dependent chromatin modification and is associated with poor prognosis in colorectal cancers. Cancer Res. American Association for Cancer Research; 2011;71:6320–6.

27. Qi P, Xu M-D, Ni S-J, Huang D, Wei P, Tan C, et al. Low expression of LOC285194 is associated with poor prognosis in colorectal cancer. J Transl Med. BioMed Central Ltd; 2013;11:122.

28. Ge X, Chen Y, Liao X, Liu D, Li F, Ruan H, et al. Overexpression of long noncoding RNA PCAT-1 is a novel biomarker of poor prognosis in patients with colorectal cancer. Med. Oncol. Springer US; 2013;30:588–6.

29. Han Y, Yang Y-N, Yuan H-H, Zhang T-T, Sui H, Wei X-L, et al. UCA1, a long non-coding RNA up-regulated in colorectal cancer influences cell proliferation, apoptosis and cell cycle distribution. Pathology. 2014;46:396–401.

30. Hu Y, Chen H-Y, Yu C-Y, Xu J, Wang J-L, Qian J, et al. A long non-coding RNA signature to improve prognosis prediction of colorectal cancer. Oncotarget. 2014;5:2230–42.

31. Chen H, Xu J, Hong J, Tang R, Zhang X, Fang J-Y. Long noncoding RNA profiles identify five distinct molecular subtypes of colorectal cancer with clinical relevance. Mol Oncol. 2014;8:1393–403.

32. Shi D, Zheng H, Zhuo C, Peng J, Li D, Xu Y, et al. Low expression of novel lncRNA RP11-462C24.1 suggests a biomarker of poor prognosis in colorectal cancer. Med. Oncol. Springer US; 2014;31:31–9.

33. He X, Bao W, Li X, Chen Z, Che Q, Wang H, et al. The long non-coding RNA HOTAIR is upregulated in endometrial carcinoma and correlates with poor prognosis. Int. J. Mol. Med. Spandidos Publications; 2014;33:325–32.

34. Zhang X, Xu Y, He C, Guo X, Zhang J, He C, et al. Elevated expression of CCAT2 is associated with poor prognosis in esophageal squamous cell carcinoma. J Surg Oncol. 2015;111:834–9.

35. Tong Y-S, Zhou X-L, Wang X-W, Wu Q-Q, Yang T-X, Lv J, et al. Association of decreased expression of long non-coding RNA LOC285194 with chemoradiotherapy resistance and poor prognosis in esophageal squamous cell carcinoma. J Transl Med. BioMed Central Ltd; 2014;12:233.

36. Pan F, Yao J, Chen Y, Zhou C, Geng P, Mao H, et al. A novel long non-coding RNA FOXCUT and mRNA FOXC1 pair promote progression and predict poor prognosis in esophageal squamous cell carcinoma. Int J Clin Exp Pathol. 2014;7:2838–49.

37. Ge X-S, Ma H-J, Zheng X-H, Ruan H-L, Liao X-Y, Xue W-Q, et al. HOTAIR, a prognostic factor in esophageal squamous cell carcinoma, inhibits WIF-1 expression and activates Wnt pathway. Cancer Sci. 2013;104:1675–82.

38. Lv X-B, Lian G-Y, Wang H-R, Song E, Yao H, Wang M-H. Long noncoding RNA HOTAIR is a prognostic marker for esophageal squamous cell carcinoma progression and survival. Aziz SA, editor. PLoS ONE. 2013;8:e63516.

39. Chen F-J, Sun M, Li S-Q, Wu Q-Q, Ji L, Liu Z-L, et al. Upregulation of the long non-coding RNA HOTAIR promotes esophageal squamous cell carcinoma metastasis and poor prognosis. Mol Carcinog. 2013;52:908–15.

40. Shi W-H, Wu Q-Q, Li S-Q, Yang T-X, Liu Z-H, Tong Y-S, et al. Upregulation of the long noncoding RNA PCAT-1 correlates with advanced clinical stage and poor prognosis in esophageal squamous carcinoma. Tumour Biol. Springer Netherlands; 2015;36:2501–7.

41. Xie H-W, Wu Q-Q, Zhu B, Chen F-J, Ji L, Li S-Q, et al. Long noncoding RNA SPRY4-IT1 is upregulated in esophageal squamous cell carcinoma and associated with poor prognosis. Tumour Biol. Springer Netherlands; 2014;35:7743–54.

42. Li J-Y, Ma X, Zhang C-B. Overexpression of long non-coding RNA UCA1 predicts a poor prognosis in patients with esophageal squamous cell carcinoma. Int J Clin Exp Pathol. 2014;7:7938–44.

43. Li J, Chen Z, Tian L, Zhou C, He MY, Gao Y, et al. LncRNA profile study reveals a three-lncRNA signature associated with the survival of patients with oesophageal squamous cell carcinoma. Gut. BMJ Publishing Group Ltd and British Society of Gastroenterology; 2014;63:1700–10.

44. Xu T-P, Huang M-D, Xia R, Liu X-X, Sun M, Yin L, et al. Decreased expression of the long non-coding RNA FENDRR is associated with poor prognosis in gastric cancer and FENDRR regulates gastric cancer cell metastasis by affecting fibronectin1 expression. J Hematol Oncol. BioMed Central Ltd; 2014;7:63.

45. Sun M, Jin F-Y, Xia R, Kong R, Li J-H, Xu T-P, et al. Decreased expression of long noncoding RNA GAS5 indicates a poor prognosis and promotes cell proliferation in gastric cancer. BMC Cancer. BioMed Central Ltd; 2014;14:319.

46. Zhang E-B, Han L, Yin D-D, Kong R, De W, Chen J. c-Myc-induced, long, noncoding H19 affects cell proliferation and predicts a poor prognosis in patients with gastric cancer. Med. Oncol. Springer US; 2014;31:914–8.

47. Chen W-M, Huang M-D, Kong R, Xu T-P, Zhang E-B, Xia R, et al. Antisense Long Noncoding RNA HIF1A-AS2 Is Upregulated in Gastric Cancer and Associated with Poor Prognosis. Dig Dis Sci. Springer US; 2015;60:1655–62.

48. Guo W, Dong Z, Bai Y, Guo Y, Shen S, Kuang G, et al. Associations between polymorphisms of HOTAIR and risk of gastric cardia adenocarcinoma in a population of north China. Tumour Biol. Springer Netherlands; 2015;36:2845–54.

49. Endo H, Shiroki T, Nakagawa T, Yokoyama M, Tamai K, Yamanami H, et al. Enhanced expression of long non-coding RNA HOTAIR is associated with the development of gastric cancer. Deng D, editor. PLoS ONE. 2013;8:e77070.

50. Sun M, Xia R, Jin F, Xu T, Liu Z, De W, et al. Downregulated long noncoding RNA MEG3 is associated with poor prognosis and promotes cell proliferation in gastric cancer. Tumour Biol. Springer Netherlands; 2014;35:1065–73.

51. Xu Z-Y, Yu Q-M, Du Y-A, Yang L-T, Dong R-Z, Huang L, et al. Knockdown of long non-coding RNA HOTAIR suppresses tumor invasion and reverses epithelial-mesenchymal transition in gastric cancer. Int J Biol Sci. 2013;9:587–97.

52. Okugawa Y, Toiyama Y, Hur K, Toden S, Saigusa S, Tanaka K, et al. Metastasis-associated long non-coding RNA drives gastric cancer development and promotes peritoneal metastasis. Carcinogenesis. Oxford University Press; 2014;35:2731–9.

53. Zhang E-B, Kong R, Yin D-D, You L-H, Sun M, Han L, et al. Long noncoding RNA ANRIL indicates a poor prognosis of gastric cancer and promotes tumor growth by epigenetically silencing of miR-99a/miR-449a. Oncotarget. 2014;5:2276–92.

54. Hu Y, Wang J, Qian J, Kong X, Tang J, Wang Y, et al. Long noncoding RNA GAPLINC regulates CD44-dependent cell invasiveness and associates with poor prognosis of gastric cancer. Cancer Res. American Association for Cancer Research; 2014;74:6890–902.

55. Yang F, Xue X, Zheng L, Bi J, Zhou Y, Zhi K, et al. Long non-coding RNA GHET1 promotes gastric carcinoma cell proliferation by increasing c-Myc mRNA stability. FEBS J. 2014;281:802–13.

56. Li H, Yu B, Li J, Su L, Yan M, Zhu Z, et al. Overexpression of lncRNA H19 enhances carcinogenesis and metastasis of gastric cancer. Oncotarget. 2014;5:2318–29.

57. Liu X-H, Sun M, Nie F-Q, Ge Y-B, Zhang E-B, Yin D-D, et al. Lnc RNA HOTAIR functions as a competing endogenous RNA to regulate HER2 expression by sponging miR-331-3p in gastric cancer. Mol Cancer. BioMed Central; 2014;13:92.

58. Wang Y, Zhang D, Wu K, Zhao Q, Nie Y, Fan D. Long noncoding RNA MRUL promotes ABCB1 expression in multidrug-resistant gastric cancer cell sublines. Mol Cell Biol. American Society for Microbiology; 2014;34:3182–93.

59. Xu M-D, Qi P, Weng W-W, Shen X-H, Ni S-J, Dong L, et al. Long non-coding RNA LSINCT5 predicts negative prognosis and exhibits oncogenic activity in gastric cancer. Medicine (Baltimore). 2014;93:e303.

60. Niinuma T, Suzuki H, Nojima M, Nosho K, Yamamoto H, Takamaru H, et al. Upregulation of miR-196a and HOTAIR drive malignant character in gastrointestinal stromal tumors. Cancer Res. American Association for Cancer Research; 2012;72:1126–36.

61. Zhang J-X, Han L, Bao Z-S, Wang Y-Y, Chen L-Y, Yan W, et al. HOTAIR, a cell cycle-associated long noncoding RNA and a strong predictor of survival, is preferentially expressed in classical and mesenchymal glioma. Neuro Oncol. Oxford University Press; 2013;15:1595–603.

62. Cho S-F, Chang YC, Chang C-S, Lin S-F, Liu Y-C, Hsiao H-H, et al. MALAT1 long non-coding RNA is overexpressed in multiple myeloma and may serve as a marker to predict disease progression. BMC Cancer. BioMed Central Ltd; 2014;14:809.

63. Esteves LICV, Javaroni AC, Nishimoto IN, Magrin J, Squire JA, Kowalski LP, et al. DNA methylation in the CTCF-binding site I and the expression pattern of the H19 gene: does positive expression predict poor prognosis in early stage head and neck carcinomas? Mol Carcinog. Wiley Subscription Services, Inc., A Wiley Company; 2005;44:102–10.

64. Tu Z-Q, Li R-J, Mei J-Z, Li X-H. Down-regulation of long non-coding RNA GAS5 is associated with the prognosis of hepatocellular carcinoma. Int J Clin Exp Pathol. 2014;7:4303–9.

65. Zhang L, Yang F, Yuan J-H, Yuan S-X, Zhou W-P, Huo X-S, et al. Epigenetic activation of the MiR-200 family contributes to H19-mediated metastasis suppression in hepatocellular carcinoma. Carcinogenesis. Oxford University Press; 2013;34:577–86.

66. Ishibashi M, Kogo R, Shibata K, Sawada G, Takahashi Y, Kurashige J, et al. Clinical significance of the expression of long non-coding RNA HOTAIR in primary hepatocellular carcinoma. Oncol Rep. Spandidos Publications; 2013;29:946–50.

67. Yang F, Zhang L, Huo X-S, Yuan J-H, Xu D, Yuan S-X, et al. Long noncoding RNA high expression in hepatocellular carcinoma facilitates tumor growth through enhancer of zeste homolog 2 in humans. Hepatology. Wiley Subscription Services, Inc., A Wiley Company; 2011;54:1679–89.

68. Quagliata L, Matter MS, Piscuoglio S, Arabi L, Ruiz C, Procino A, et al. Long noncoding RNA HOTTIP/HOXA13 expression is associated with disease progression and predicts outcome in hepatocellular carcinoma patients. Hepatology. 2014;59:911–23.

69. Yang Z, Zhou L, Wu L-M, Lai M-C, Xie H-Y, Zhang F, et al. Overexpression of long non-coding RNA HOTAIR predicts tumor recurrence in hepatocellular carcinoma patients following liver transplantation. Ann Surg Oncol. Springer-Verlag; 2011;18:1243–50.

70. Lai M-C, Yang Z, Zhou L, Zhu Q-Q, Xie H-Y, Zhang F, et al. Long non-coding RNA MALAT-1 overexpression predicts tumor recurrence of hepatocellular carcinoma after liver transplantation. Med. Oncol. Springer US; 2012;29:1810–6.

71. Yuan S-X, Yang F, Yang Y, Tao Q-F, Zhang J, Huang G, et al. Long noncoding RNA associated with microvascular invasion in hepatocellular carcinoma promotes angiogenesis and serves as a predictor for hepatocellular carcinoma patients' poor recurrence-free survival after hepatectomy. Hepatology. Wiley Subscription Services, Inc., A Wiley Company; 2012;56:2231–41.

72. Wang F, Yuan J-H, Wang S-B, Yang F, Yuan S-X, Ye C, et al. Oncofetal long noncoding RNA PVT1 promotes proliferation and stem cell-like property of hepatocellular carcinoma cells by stabilizing NOP2. Hepatology. 2014;60:1278–90.

73. Fellig Y, Ariel I, Ohana P, Schachter P, Sinelnikov I, Birman T, et al. H19 expression in hepatic metastases from a range of human carcinomas. J Clin Pathol. BMJ Publishing Group Ltd and Association of Clinical Pathologists; 2005;58:1064–8.

74. Li D, Feng J, Wu T, Wang Y, Sun Y, Ren J, et al. Long intergenic noncoding RNA HOTAIR is overexpressed and regulates PTEN methylation in laryngeal squamous cell carcinoma. Am J Pathol. 2013;182:64–70.

75. Garzon R, Volinia S, Papaioannou D, Nicolet D, Kohlschmidt J, Yan PS, et al. Expression and prognostic impact of lncRNAs in acute myeloid leukemia. Proc Natl Acad Sci U S A. National Acad Sciences; 2014;111:18679–84.

76. Sun M, Liu X-H, Wang K-M, Nie F-Q, Kong R, Yang J-S, et al. Downregulation of BRAF activated non-coding RNA is associated with poor prognosis for non-small cell lung cancer and promotes metastasis by affecting epithelial-mesenchymal transition. Mol Cancer. BioMed Central; 2014;13:68.

77. Zhang L, Zhou X-F, Pan G-F, Zhao J-P. Enhanced expression of long non-coding RNA ZXF1 promoted the invasion and metastasis in lung adenocarcinoma. Biomed Pharmacother. 2014;68:401–7.

78. Luo J, Tang L, Zhang J, Ni J, Zhang H-P, Zhang L, et al. Long non-coding RNA CARLo-5 is a negative prognostic factor and exhibits tumor pro-oncogenic activity in non-small cell lung cancer. Tumour Biol. Springer Netherlands; 2014;35:11541–9.

79. Han L, Kong R, Yin D-D, Zhang E-B, Xu T-P, De W, et al. Low expression of long noncoding RNA GAS6-AS1 predicts a poor prognosis in patients with NSCLC. Med. Oncol. Springer US; 2013;30:694–7.

80. Nakagawa T, Endo H, Yokoyama M, Abe J, Tamai K, Tanaka N, et al. Large noncoding RNA HOTAIR enhances aggressive biological behavior and is associated with short disease-free survival in human non-small cell lung cancer. Biochem Biophys Res Commun. 2013;436:319–24.

81. Liu X-H, Liu Z-L, Sun M, Liu J, Wang Z-X, De W. The long non-coding RNA HOTAIR indicates a poor prognosis and promotes metastasis in non-small cell lung cancer. BMC Cancer. BioMed Central Ltd; 2013;13:464.

82. Schmidt LH, Spieker T, Koschmieder S, Schaffers S, Humberg J, Jungen D, et al. The long noncoding MALAT-1 RNA indicates a poor prognosis in non-small cell lung cancer and induces migration and tumor growth. J Thorac Oncol. 2011;6:1984–92.

83. Lu K-H, Li W, Liu X-H, Sun M, Zhang M-L, Wu W-Q, et al. Long non-coding RNA MEG3 inhibits NSCLC cells proliferation and induces apoptosis by affecting p53 expression. BMC Cancer. BioMed Central Ltd; 2013;13:461.

84. Nie F-Q, Zhu Q, Xu T-P, Zou Y-F, Xie M, Sun M, et al. Long non-coding RNA MVIH indicates a poor prognosis for non-small cell lung cancer and promotes cell proliferation and invasion. Tumour Biol. Springer Netherlands; 2014;35:7587–94.

85. Yang Y-R, Zang S-Z, Zhong C-L, Li Y-X, Zhao S-S, Feng X-J. Increased expression of the lncRNA PVT1 promotes tumorigenesis in non-small cell lung cancer. Int J Clin Exp Pathol. 2014;7:6929–35.

86. Hou Z, Zhao W, Zhou J, Shen L, Zhan P, Xu C, et al. A long noncoding RNA Sox2ot regulates lung cancer cell proliferation and is a prognostic indicator of poor survival. Int J Biochem Cell Biol. 2014;53:380–8.

87. Zhang E-B, Yin D-D, Sun M, Kong R, Liu X-H, You L-H, et al. P53-regulated long non-coding RNA TUG1 affects cell proliferation in human non-small cell lung cancer, partly through epigenetically regulating HOXB7 expression. Cell Death Dis. 2014;5:e1243.

88. Shen L, Chen L, Wang Y, Jiang X, Xia H, Zhuang Z. Long noncoding RNA MALAT1 promotes brain metastasis by inducing epithelial-mesenchymal transition in lung cancer. J. Neurooncol. Springer US; 2015;121:101–8.

89. Ono H, Motoi N, Nagano H, Miyauchi E, Ushijima M, Matsuura M, et al. Long noncoding RNA HOTAIR is relevant to cellular proliferation, invasiveness, and clinical relapse in small-cell lung cancer. Cancer Med. 2014;3:632–42.

90. Du Z, Fei T, Verhaak RGW, Su Z, Zhang Y, Brown M, et al. Integrative genomic analyses reveal clinically relevant long noncoding RNAs in human cancer. Nat Struct Mol Biol. 2013;20:908–13.

91. Zhang W, Huang C, Gong Z, Zhao Y, Tang K, Li X, et al. Expression of LINC00312, a long intergenic non-coding RNA, is negatively correlated with tumor size but positively correlated with lymph node metastasis in nasopharyngeal carcinoma. J Mol Histol. Springer Netherlands; 2013;44:545–54.

92. Nie Y, Liu X, Qu S, Song E, Zou H, Gong C. Long non-coding RNA HOTAIR is an independent prognostic marker for nasopharyngeal carcinoma progression and survival. Cancer Sci. 2013;104:458–64.

93. Barnhill LM, Williams RT, Cohen O, Kim Y, Batova A, Mielke JA, et al. High expression of CAI2, a 9p21-embedded long noncoding RNA, contributes to advanced-stage neuroblastoma. Cancer Res. American Association for Cancer Research; 2014;74:3753–63.

94. Qiu J-J, Ye L-C, Ding J-X, Feng W-W, Jin H-Y, Zhang Y, et al. Expression and clinical significance of estrogen-regulated long non-coding RNAs in estrogen receptor alpha-positive ovarian cancer progression. Oncol Rep. Spandidos Publications; 2014;31:1613–22.

95. Qiu J-J, Lin Y-Y, Ye L-C, Ding J-X, Feng W-W, Jin H-Y, et al. Overexpression of long non-coding RNA HOTAIR predicts poor patient prognosis and promotes tumor metastasis in epithelial ovarian cancer. Gynecol Oncol. 2014;134:121–8.

96. Qiu J-J, Wang Y, Ding J-X, Jin H-Y, Yang G, Hua K-Q. The long non-coding RNA HOTAIR promotes the proliferation of serous ovarian cancer cells through the regulation of cell cycle arrest and apoptosis. Exp Cell Res. 2015;333:238–48.

97. Liu J-H, Chen G, Dang Y-W, Li C-J, Luo D-Z. Expression and prognostic significance of lncRNA MALAT1 in pancreatic cancer tissues. Asian Pac J Cancer Prev. 2014;15:2971–7.

98. Huang C, Yu W, Wang Q, Cui H, Wang Y, Zhang L, et al. Increased expression of the lncRNA PVT1 is associated with poor prognosis in pancreatic cancer patients. Minerva Med. 2015;106:143–9.

99. Pang E-J, Yang R, Fu X-B, Liu Y-F. Overexpression of long non-coding RNA MALAT1 is correlated with clinical progression and unfavorable prognosis in pancreatic cancer. Tumour Biol. Springer Netherlands; 2015;36:2403–7.

100. Sun Y-W, Chen Y-F, Li J, Huo Y-M, Liu D-J, Hua R, et al. A novel long non-coding RNA ENST00000480739 suppresses tumour cell invasion by regulating OS-9 and HIF-1alpha in pancreatic ductal adenocarcinoma. Br J Cancer. 2014;111:2131–41.

101. Ding Y-C, Yu W, Ma C, Wang Q, Huang C-S, Huang T. Expression of long non-coding RNA LOC285194 and its prognostic significance in human pancreatic ductal adenocarcinoma. Int J Clin Exp Pathol. 2014;7:8065–70.

102. Li J, Liu D, Hua R, Zhang J, Liu W, Huo Y, et al. Long non-coding RNAs expressed in pancreatic ductal adenocarcinoma and lncRNA BC008363 an independent prognostic factor in PDAC. Pancreatology. 2014;14:385–90.

103. Prensner JR, Sahu A, Iyer MK, Malik R, Chandler B, Asangani IA, et al. The IncRNAs PCGEM1 and PRNCR1 are not implicated in castration resistant prostate cancer. Oncotarget. 2014;5:1434–8.

104. Mehra R, Shi Y, Udager AM, Prensner JR, Sahu A, Iyer MK, et al. A novel RNA in situ hybridization assay for the long noncoding RNA SChLAP1 predicts poor clinical outcome after radical prostatectomy in clinically localized prostate cancer. Neoplasia. 2014;16:1121–7.

105. Malik R, Patel L, Prensner JR, Shi Y, Iyer MK, Subramaniyan S, et al. The lncRNA PCAT29 inhibits oncogenic phenotypes in prostate cancer. Mol Cancer Res. American Association for Cancer Research; 2014;12:1081–7.

106. Prensner JR, Iyer MK, Sahu A, Asangani IA, Cao Q, Patel L, et al. The long noncoding RNA SChLAP1 promotes aggressive prostate cancer and antagonizes the SWI/SNF complex. Nat. Genet. 2013;45:1392–8.

107. Prensner JR, Zhao S, Erho N, Schipper M, Iyer MK, Dhanasekaran SM, et al. RNA biomarkers associated with metastatic progression in prostate cancer: a multi-institutional high-throughput analysis of SChLAP1. Lancet Oncol. 2014;15:1469–80.

108. Yao J, Chen Y, Wang Y, Liu S, Yuan X, Pan F, et al. Decreased expression of a novel lncRNA CADM1-AS1 is associated with poor prognosis in patients with clear cell renal cell carcinomas. Int J Clin Exp Pathol. 2014;7:2758–67.

109. Zhang H-M, Yang F-Q, Yan Y, Che J-P, Zheng J-H. High expression of long non-coding RNA SPRY4-IT1 predicts poor prognosis of clear cell renal cell carcinoma. Int J Clin Exp Pathol. 2014;7:5801–9.

110. Zhang H-M, Yang F-Q, Chen S-J, Che J, Zheng J-H. Upregulation of long non-coding RNA MALAT1 correlates with tumor progression and poor prognosis in clear cell renal cell carcinoma. Tumour Biol. Springer Netherlands; 2015;36:2947–55.

111. Song S, Wu Z, Wang C, Liu B, Ye X, Chen J, et al. RCCRT1 is correlated with prognosis and promotes cell migration and invasion in renal cell carcinoma. Urology. 2014;84:730.e1–7.
