# Supplementary material for: Long noncoding RNAs as novel predictors of survival in human cancer: a systematic review and meta-analysis
Source: Mol Cancer. 2016 Jun 28;15:50. doi: 10.1186/s12943-016-0535-1 (PMC4924330; doi:10.1186/s12943-016-0535-1)
Supplement: Additional file 3: Figure S1. — The covariates included within the multivariable models fitted by each paper. This is a data microarray in which the studies run along the Y-axis and the covariates run along the X-axis. Rows and columns are ordered in descending order, based on the total number each covariate was included in the multivariable models fitted by each study. Where patterns were similar between studies or covariates, those studies or covariates were placed next to each other. (PDF 82 kb) [file 12943_2016_535_MOESM3_ESM.pdf]

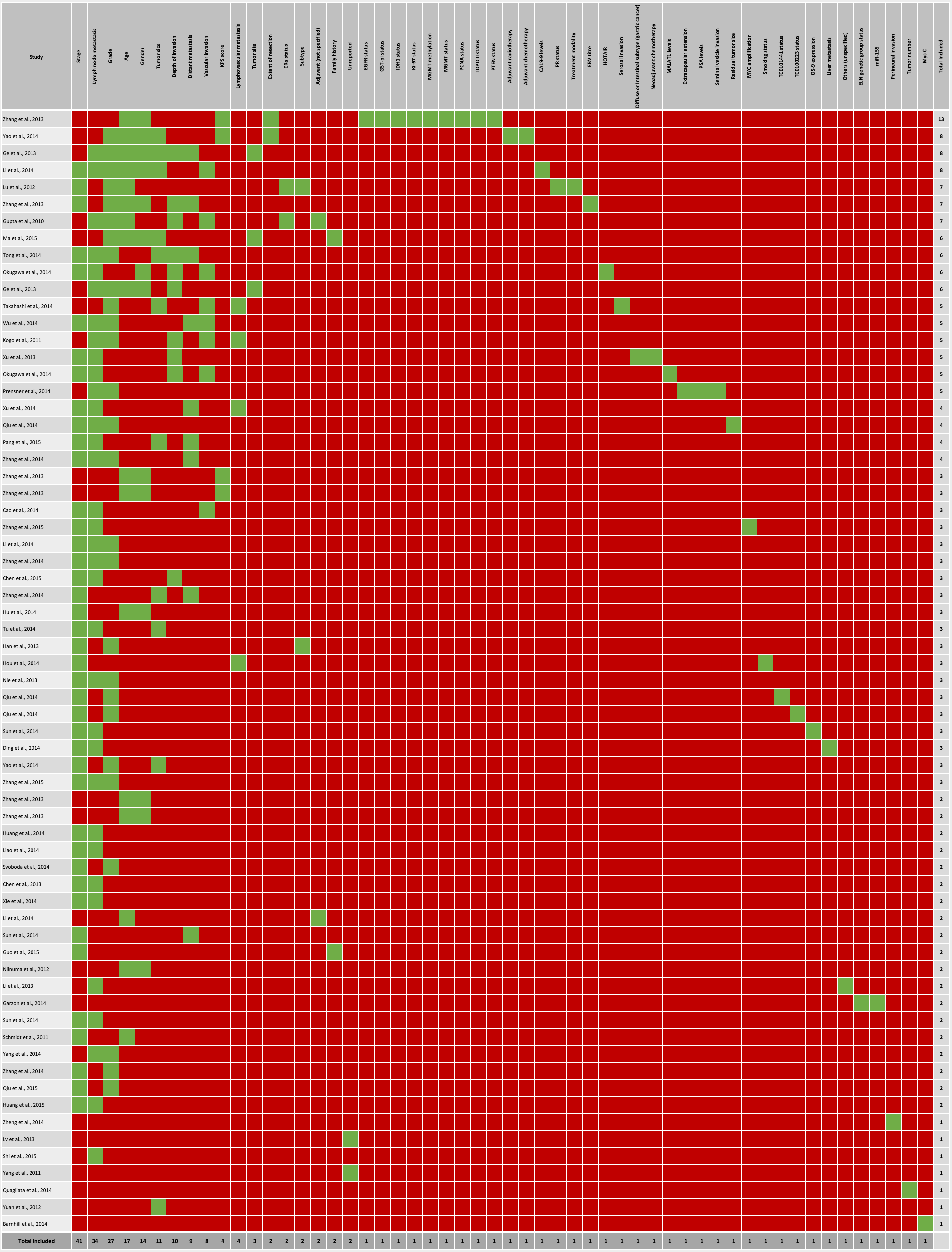
Study
Stage
Lymph node metastasis
Grade
Age
Gender
Tumor size
Depth of invasion
Distant metastasis
Vascular invasion
KPS score
Lymphovascular metastasis
Tumor site
Extent of resection
ERa status
Subtype
Adjuvant (not specified)
Family history
Unreported
EGFR status
GST-pi status
IDH1 status
Ki-67 status
MGMT methylation
MGMT status
PCNA status
TOPO II status
PTEN status
Adjuvant radiotherapy
Adjuvant chemotherapy
CA19-9 levels
PR status
Treatment modality
EBV titre
HOTAIR
Serosal invasion
Diffuse or Intestinal subtype (gastric cancer)
Neoadjuvant chemotherapy
MALAT1 levels
Extracapsular extension
PSA levels
Seminal vesicle invasion
Residual tumor size
MYC amplification
Smoking status
TC0101441 status
TC0100223 status
OS-9 expression
Liver metastasis
Others (unspecified)
ELN genetic group status
miR-155
Perineural invasion
Tumor number
Myc C
Total Included
Zhang et al., 2013 13
Yao et al., 2014 8
Ge et al., 2013 8
Li et al., 2014 8
Lu et al., 2012 7
Zhang et al., 2013 7
Gupta et al., 2010 7
Ma et al., 2015 6
Tong et al., 2014 6
Okugawa et al., 2014 6
Ge et al., 2013 6
Takahashi et al., 2014 5
Wu et al., 2014 5
Kogo et al., 2011 5
Xu et al., 2013 5
Okugawa et al., 2014 5
Prensner et al., 2014 5
Xu et al., 2014 4
Qiu et al., 2014 4
Pang et al., 2015 4
Zhang et al., 2014 4
Zhang et al., 2013 3
Zhang et al., 2013 3
Cao et al., 2014 3
Zhang et al., 2015 3
Li et al., 2014 3
Zhang et al., 2014 3
Chen et al., 2015 3
Zhang et al., 2014 3
Hu et al., 2014 3
Tu et al., 2014 3
Han et al., 2013 3
Hou et al., 2014 3
Nie et al., 2013 3
Qiu et al., 2014 3
Qiu et al., 2014 3
Sun et al., 2014 3
Ding et al., 2014 3
Yao et al., 2014 3
Zhang et al., 2015 3
Zhang et al., 2013 2
Zhang et al., 2013 2
Huang et al., 2014 2
Liao et al., 2014 2
Svoboda et al., 2014 2
Chen et al., 2013 2
Xie et al., 2014 2
Li et al., 2014 2
Sun et al., 2014 2
Guo et al., 2015 2
Niinuma et al., 2012 2
Li et al., 2013 2
Garzon et al., 2014 2
Sun et al., 2014 2
Schmidt et al., 2011 2
Yang et al., 2014 2
Zhang et al., 2014 2
Qiu et al., 2015 2
Huang et al., 2015 2
Zheng et al., 2014 1
Lv et al., 2013 1
Shi et al., 2015 1
Yang et al., 2011 1
Quagliata et al., 2014 1
Yuan et al., 2012 1
Barnhill et al., 2014 1
Total Included 41 34 27 17 14 11 10 9 8 4 4 3 2 2 2 2 2 2 1 1 1 1 1 1 1 1 1 1 1 1 1 1 1 1 1 1 1 1 1 1 1 1 1 1 1 1 1 1 1 1 1 1 1 1

Figure S1. The covariates included within the multivariable models fitted by each paper. This is a data microarray in which the studies run along the Y-axis and the covariates run along the X-axis. Rows and columns are ordered in descending order, based on the total number each covariate was included in the multivariable models fitted by each study. Where patterns were similar between studies or covariates, those studies or covariates were placed next to each other.

It is evident that very few studies included the same covariates within their models and that less than half of the studies included both Stage and Grade within those models. Interestingly, the majority of studies included at least one covariate within their model that had not been included in any other study. Green = Included in the multivariable model; Red = Not included in the multivariable model.
