# Supplementary material for: Long noncoding RNAs as novel predictors of survival in human cancer: a systematic review and meta-analysis
Source: Mol Cancer. 2016 Jun 28;15:50. doi: 10.1186/s12943-016-0535-1 (PMC4924330; doi:10.1186/s12943-016-0535-1)
Supplement: Additional file 4: Table S1. — Explanation of how 92 studies provided data regarding 127 analyses. (DOC 36 kb) [file 12943_2016_535_MOESM4_ESM.doc]

## Table S1

**Table S1.** Explanation of how 92 studies provided data regarding 127 analyses.

| **Study** | **Analysis datasets** | **Explanation** |
| --- | --- | --- |
| Wang et al., 2015 [2] | 5 | 6 different lncRNAs |
| Zhang et al., 2013 [7] | 10 | Studied the 6-lncRNA score in 3 different cohorts + for one of the cohorts (GSE7696) they used two mutually exclusive subtypes (treatment with radiotherapy, treatment with radiotherapy + chemotherapy) + separately associated each lncRNA of the 6-lncRNA risk score to survival |
| Li et al., 2014 [10] | 2 | 2 different datasets |
| Endo et al., 2013 [49] | 2 | 2 mutually exclusive subtypes of gastric cancer (intestinal vs diffuse) |
| Okugawa et al., 2014 [52] | 4 | 2 lncRNAs studied in all gastric cancers + the same 2 lncRNAs were also studied in those gastric cancers with peritoneal metastasis |
| Zhang et al., 2013 [61] | 7 | 3 different cohorts - in the 1st AND 3rd cohorts there was 1 analysis of high grade glioma that was then sub-classified into 1 anaplastic and 1 glioblastoma multiforme (GBM) analysis + 1 analysis of GBM in a second cohort |
| Quagliata et al., 2014 [68] | 2 | 2 different lncRNAs |
| Yuan et al., 2012 [71] | 2 | 1 study of all hepatocellular carcinoma (HCC) + 1 study of early HCC |
| Schmidt et al., 2011 [82] | 2 | 2 mutually exclusive subtypes (non-small cell lung cancer (NSCLC) squamous cell carcinoma (SCC) vs NSCLC non-SCC) |
| Du et al., 2013 [90] | 2 | 2 different cancers |
| Zhang et al., 2013 [91] | 3 | 1 analysis of naropharyngeal cancer without sub-classifications + 2 mutually exclusive sub-classifications (with lymph node metastasis (LNM) vs without LNM) |
| Barnhill et al., 2014 [93] | 2 | 1 study of all neuroblastoma + 1 of only late stage neuroblastoma |
| Qiu et al., 2014 [94] | 3 | 3 different lncRNAs |
| Prensner et al., 2013 [106] | 2 | 2 different datasets |
| Prensner et al., 2014 [107] | 2 | 1 complete dataset + 1 non-mutually exclusive sub-dataset |
